# Finite Element Simulations of the ID Venous System to Treat Venous Compression Disorders: From Model Validation to Realistic Implant Prediction

**DOI:** 10.1007/s10439-020-02694-8

**Published:** 2021-01-04

**Authors:** Alissa Zaccaria, Francesco Migliavacca, David Contassot, Frederic Heim, Nabil Chakfe, Giancarlo Pennati, Lorenza Petrini

**Affiliations:** 1 LaBS, Department of Chemistry Materials and Chemical Engineering, Politecnico di Milano, Milan, Italy; 2 ID NEST MEDICAL SAS, Strasbourg, France; 3 Laboratoire de Physique et Mécanique Textiles (LPMT), Université de Haute-Alsace, Mulhouse, France; 4 Groupe Européen De Recherche Sur Les Prothèses Appliquées À La Chirurgie Vasculaire (GEPROVAS), Strasbourg, France; 5 Hôpitaux Universitaires de Strasbourg, Strasbourg, France; 6 Department of Civil and Environmental Engineering, Politecnico di Milano, Milano, Italy

**Keywords:** May-Thurner syndrome, Venous stent, Bifurcation, *In-silico* modeling, *In-vitro* testing, Nitinol, Self-expandable stent

## Abstract

The ID Venous System is an innovative device proposed by ID NEST MEDICAL to treat venous compression disorders that involve bifurcations, such as the May-Thurner syndrome. The system consists of two components, ID Cav and ID Branch, combined through a specific connection that prevents the migration acting locally on the pathological region, thereby preserving the surrounding healthy tissues. Preliminary trials are required to ensure the safety and efficacy of the device, including numerical simulations. *In-silico* models are intended to corroborate experimental data, providing additional local information not acquirable by other means. The present work outlines the finite element model implementation and illustrates a sequential validation process, involving seven tests of increasing complexity to assess the impact of each numerical uncertainty separately. Following the standard ASME V&V40, the computational results were compared with experimental data in terms of force-displacement curves and deformed configurations, testing the model reliability for the intended context of use (differences < 10%). The deployment in a realistic geometry confirmed the feasibility of the implant procedure, without risk of rupture or plasticity of the components, highlighting the potential of the present technology.

**Electronic supplementary material:**

The online version of this article (10.1007/s10439-020-02694-8) contains supplementary material, which is available to authorized users.

## Introduction

**Figure 1:**
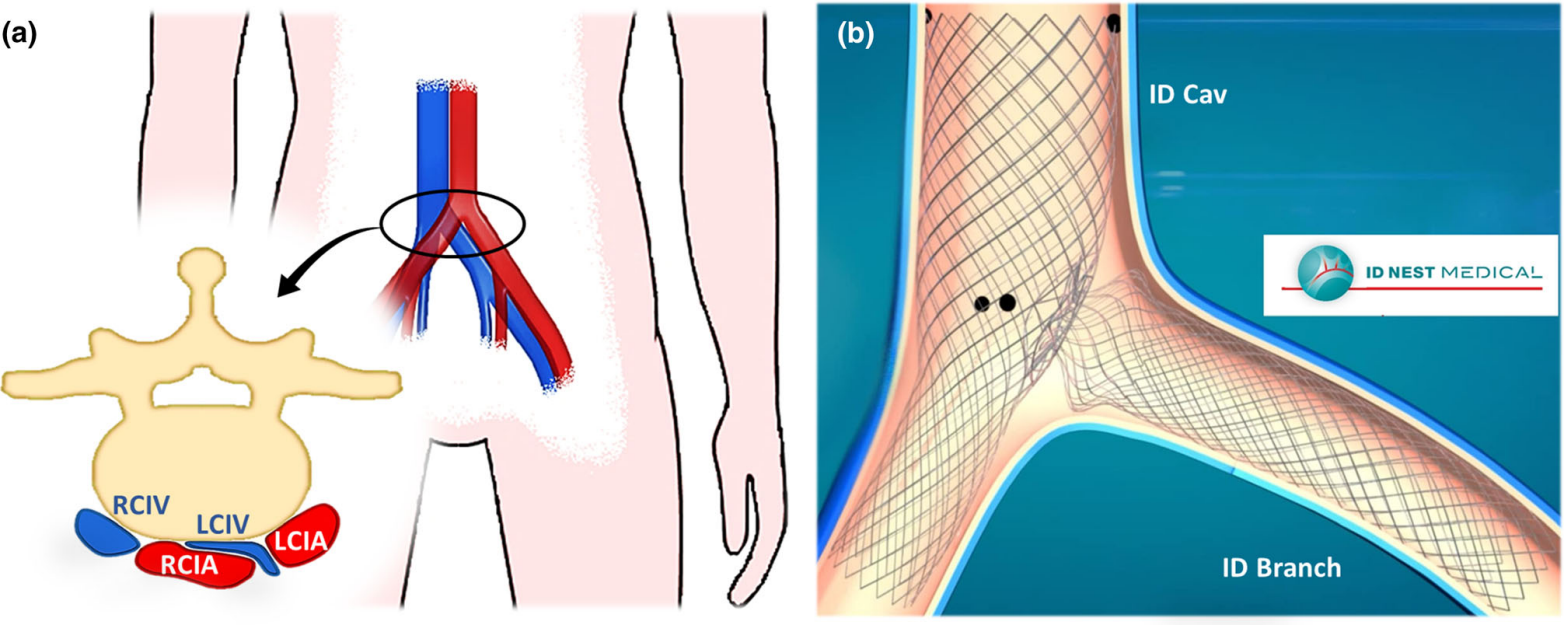
(a) Representation of the May-Thurner syndrome: compression of the left common iliac vein (LCIV) between the right common iliac artery (RCIA) and the lumbosacral spine. (b) Deployed configuration of the ID Venous System: the ID Branch is placed in the LCIV and connected to the fenestration of the ID Cav at the junction between the right common iliac vein (RCIV) and the inferior vena cava.

Nowadays, peripheral vascular diseases are widespread worldwide, affecting the quality of life of more than 200 million people, with a significant socioeconomic impact.^24^ Pathologies related to the venous system alone require the 1–2.5% of the budget allocated for the health care system in developed countries.^18^ One of the causes of chronic venous disorders is the May-Thurner syndrome, which consists of the compression of the left common iliac vein (LCIV) between the right common iliac artery (RCIA) and the lumbosacral spine (Fig. 1a). The real incidence of this pathology is unknown since most of the patients are asymptomatic. However, in severe cases, it can cause swelling and pain in the extremities, and its prevalence ranges from 18 to 49% in patients with deep vein thrombosis (DVT).^17^ Indeed, besides the chronic compression, the pulsatile force applied by the iliac artery exacerbates the stress undergone by the vein leading to local intimal hyperplasia, which increases the risk of acute and chronic DVT.^14^,^26^ The current standard treatment involves thrombolysis and iliac vein stenting. In fact, given the characteristics of the pathology, angioplasty itself is not successful.^22^ To date, only two dedicated devices have been approved by the Food and Drug Administration (FDA), namely the Venovo (BD Interventional) and Vici (Boston Scientific Corporation) device.^21^ Both of which are nitinol cylindrical stents obtained through laser-cut procedure, to avoid foreshortening and weakness in the extremities, connected with braided design. The most common diameter sizes are 14–16 mm, and their length can reach 120–160 mm, following the need to anchor the devices distally in healthy veins. In fact, isolated stents less than 80-100 mm long may result in higher migration and embolization rates, particularly in patients with post-thrombotic syndrome presenting long pathological traits.^21^

ID NEST MEDICAL (Strasbourg, France) presented an innovative system, named ID Venous System, consisting of two interacting stents, namely ID Cav and ID Branch, which prevents migration acting locally on the pathological region, due to the specifically designed accommodating connection. More precisely, the ID Cav is placed distally in the right common iliac vein (RCIV) and proximally in the inferior vena cava and features a fenestration at the inlet of the LCIV to which the ID Branch is connected. While the former is a laser cut frame with a fenestration on which a flat spring (diaphragm) is fitted, the latter is a braided structure with the proximal extreme that works like a compliant clip and incorporates double twisted traits to improve the radial rigidity in the compressed vein section (Fig. 1b). Double twisted traits limit the foreshortening and the looped ends prevent the collapse of the extremities. Consequently, the ID Venous System features great flexibility and low invasiveness, with limited disadvantages related to the braided design. Furthermore, the compliant clip is an innovative solution that differs from bifurcated devices proposed so far to treat vascular bifurcations^6^ (abdominal stent-graft, 3D printed devices^10^) and different from current implant techniques to couple stents (double D and kissing stent-grafts deployments,^4^,^7^ disconnected stents and T-stenting^25^).

The illustrated technology is still in the development process. Computational analysis can support the current design stage by investigating the effectiveness and reliability of the system and evaluating possible modifications before proceeding with *in-vivo* studies. In literature, finite element (FE) models have been widely exploited to evaluate biomedical device behavior, including endovascular prosthesis, investigating the performances of the stents and their impact on surrounding tissues.^15^,^19^ Numerical methods have also been used to investigate other previously mentioned branched stent technologies.^8^,^20^ Usually, cubic or beam elements are used to model the stent while external surfaces are exploited to mimic the crimping, positioning and release in a vessel, defined through shell or cubic elements.

As already stated in the literature, computational models need to be implemented following a rigorous verification and validation process to establish their reliability.^1^,^9^,^13^,^23^ However, only qualitative indications about the procedure to be followed have been provided,^2^ and, starting from these guidelines, for each device, it is necessary to implement an ad hoc protocol in light of the intended use and the possible impact of the model.^3^ In this specific case, *in-silico* tests provide data that, combined with *in-vitro* and in-vivo results, will be used to assess the safety and efficacy of the ID Venous System. Following the standard ASME V&V 40^3^, it is possible to assume a model risk of level 3. Indeed, the impact of the model is low, since the decision process will account for other evidence provided by in-vitro and in-vivo tests, but relevant risks are related to incorrect judgments, possibly resulting in patient injuries and increased costs. The identified risk level requires the validation through comparisons with experimental tests, and the differences with the comparator should be below 10%.

The present work aims to investigate the venous system implant feasibility through *in-silico* simulations, deepening the information provided by in-vitro tests. To this purpose, an operating proposal extendable to different stent-like devices to accomplish the definition and validation of the numerical model, starting from data provided by the manufacturer, was defined. Discussed below is the geometry reconstruction, its discretization and defining the material properties. Then, the validation process is outlined, wherein experimental tests were used as a comparator to assess the credibility of the numerical model. The tests were designed in ascending order of complexity, sequentially introducing the interactions among the components involved in order to identify possible uncertainties concerning the intended context of use. Radial and tensile tests on the two components were initially performed individually, followed by a test for the evaluation of the connection stiffness and then free release tests. Finally, the deployment in a bench test, resembling the venous bifurcation, was performed both experimentally and numerically. This was used to prove the effectiveness and reliability of the ID Venous System implant, assessing eventual system imperfections and possible refinements to be addressed before proceeding with *in-vivo* studies.

## Materials and Methods

This section illustrates

1. Finite element model development;
2. *In-silico* model validation through comparison with experiments regarding the single components (ID Cav, ID Branch and diaphragm), the assembled system (ID Branch connected with the ID Cav through the diaphragm) and the release procedure;
3. Assessment of the model capability to predict the device clinical performance by comparing *in-vitro* and *in-silico* deployment in a realistic geometry.

In total, four commercial software programs were used. The components were drawn in Solidworks (Dassault Systèmes) and discretized by Altair HyperMesh 2019 (Altair Engineering). ANSA Pre Processor v19.0 (BETA CAE Systems) was used to obtain the mesh of the bench test. For both the validation and deployment steps, all the simulations were carried out in Abaqus/Explicit 2019 (Dassault Systèmes). The displacements and loads defined were applied following smooth-step amplitudes. Moreover, the mass-scaling factor was introduced to optimize the required computational time. In each simulation, it was verified that the kinetic energy was below 5% of the internal energy throughout most of the process.

### Finite Element Model Development

The numerical model was developed according to the following steps:

1. Geometry (shape reconstruction);
2. Mesh (geometry discretization);
3. Material (defining model and parameters);
4. Interactions (defining contact properties).

#### Geometry

**Figure 2:**
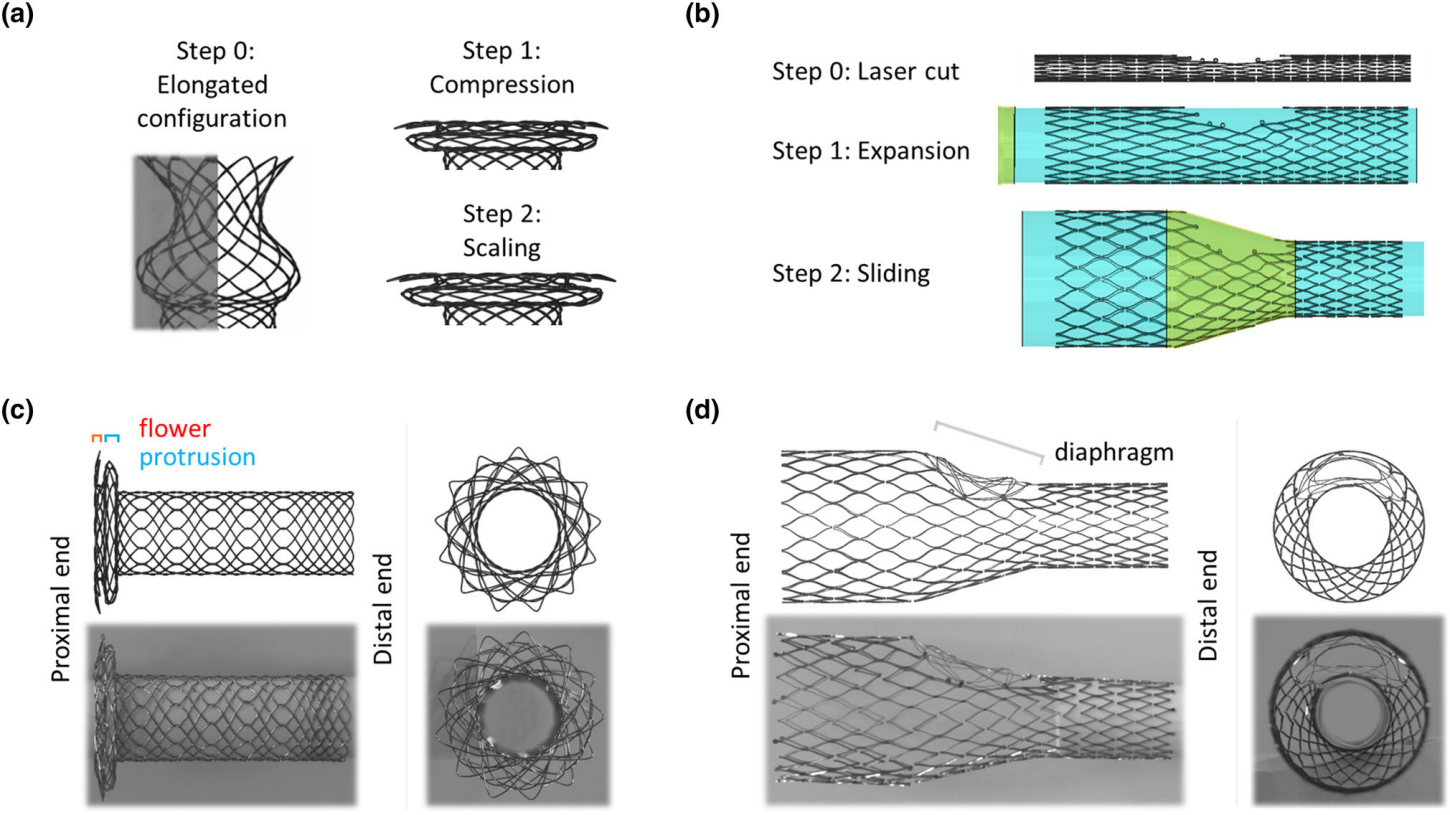
(a) ID Branch and (b) ID Cav model implementation subdivided into three main steps: extended configuration reconstruction, compression simulation, and morphing for the ID Branch; crimped configuration reconstruction, expansion of the distal and proximal trait for the ID Cav. Comparison of (c) ID Branch and (d) ID Cav model with real devices.

The ID Branch was built using 3D parametric equations.^27^ The geometrical parameters were evaluated based on pictures of the stent in the extended configuration, in order to improve the similarity with the real device. Then, a preliminary compression simulation was carried out to achieve the original shape (Fig. 2a, Step 1). However, this procedure implied some inaccuracies that resulted in incorrect protrusion and flower diameters. Thus, a further step was introduced (Fig. 2a, Step 2 “scaling”) where the coordinates of the nodes defining these two portions were scaled up to obtain the correct dimensions.

The ID Cav geometry was built starting from the drawing used during the laser-cut procedure. The section dimensions were taken from microscope images of the components after electropolishing (Online Appendix B). An expansion simulation was performed to achieve the final shape: a dedicated surface was used to initially expand the structure up to the diameter of the distal part, and then to enlarge the proximal trait, imposing a sliding feature along the surface. Finally, the diaphragm was defined based on images and the expanded ID Cav model (Fig. 2b). In Figs. 2c and 2d the final stress-free numerical models are compared with the real devices.

#### Mesh

After a mesh sensitivity analysis (Online Appendix A) the components were discretized with linear beam elements (B31) given the greater efficiency with respect to 3D elements^11^ (5548, 9243, and 522 elements were used to model the ID Branch, ID Cav, and diaphragm, respectively).

#### Materials

All the components are made of Nitinol, featuring a super-elastic behavior at the temperatures of interest (T ≥ 25 °C). The constitutive model available in Abaqus was adopted to describe the stress-strain relationship. The material parameters were calibrated based on tensile tests performed on samples provided by the manufacturer (Online Appendix B).

#### Interactions

The interaction among the system components was described with the general contact algorithm, imposing a friction coefficient equal to 0. 2.^16^

### Finite Element Model Validation

The validation process aims to prove the credibility of the model for the intended use. In this case, the simulations must reliably predict the deployment of the system and its efficacy once implanted *in situ*. It is possible to recognize three main issues to assess:

1. individual components behavior;
2. assembled system behavior;
3. release procedure.

**Figure 3:**
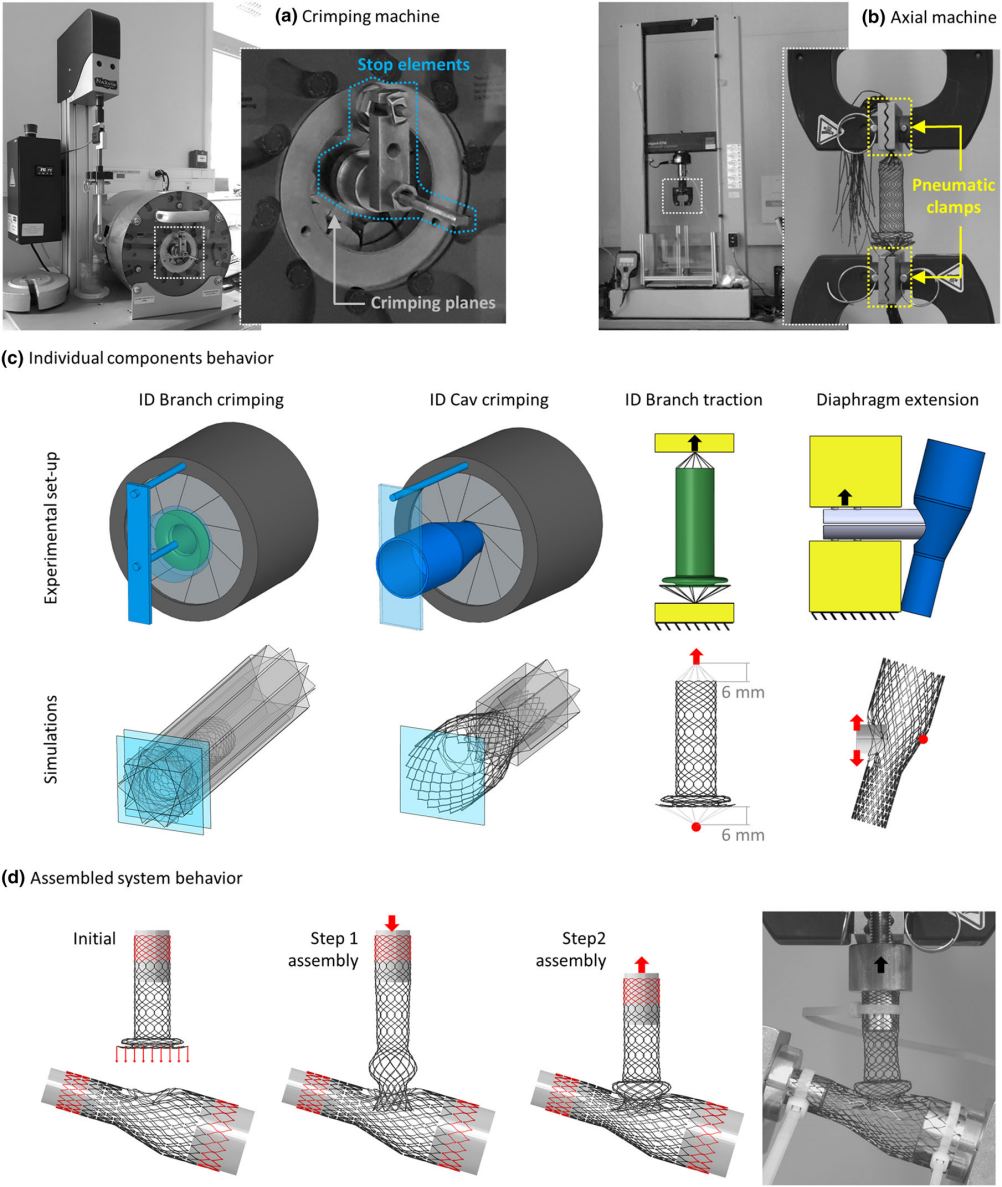
(a) Picture of the crimping machine (Blockwise Testing Machine, model TTR2 with large twin-cam™ compression station, Blockwise Engineering LLC, Tempe, AZ, USA), with on the right a magnification of the crimping planes and the stop elements highlighted in cyan. (b) Picture of the axial machine (MTS Insight Electromechanical Testing Systems, Twin-column 50 kN, MTS Systems Corporation, Eden Prairie, MN, USA), with on the right a magnification of the pneumatic clamps highlighted in yellow. (c) Individual components behavior validation tests, experimental set-up illustration, and numerical boundary conditions for the: ID Branch and ID Cav crimping, ID Branch traction, and diaphragm extension. (d) Assembled system behavior validation test: computational steps for connecting the components and obtaining the initial condition of the axial extraction test (left), and comparison with the initial experimental configuration (right).

The experimental data, to be used for comparison, was obtained performing crimping and axial tests, with an ad-hoc experimental set-up, using a Blockwise Testing Machine (model TTR2 with large twin-cam™ compression station, Blockwise Engineering LLC) and an MTS Insight Electromechanical Testing Systems (Twin-column 50 kN, MTS Systems Corporation), illustrated in Figs. 3a and 3b, respectively. Each experiment was repeated on three different samples to ensure the test repeatability. To compare experimental and numerical results, force-displacement curves and, when possible, deformed configurations were considered.

#### Individual Components Behavior

The most critical features for each device are the radial stiffness, defining the pressure acting on the vessel, and the properties influencing the connection behavior, namely the axial stiffness of the ID Branch and the Diaphragm rigidity. According to these considerations, the following tests on the two individual components were designed:

- ID Branch crimping: radial compression of the section with a constant diameter (excluding flower and protrusion, Fig. 2c) from 14 mm to 4 mm at both 25 and 37 °C;
- ID Cav crimping: radial compression of the distal trait from 15 to 4 mm at both 25 and 37 °C;
- traction of the ID Branch, connecting the stent extremities to the testing machine clamps with inextensible wires, at a distance of 6 mm (Fig. 3c), and imposing a total elongation of 70 mm;
- extension of the diaphragm, using two semi-cylindrical bars (12 mm diameter) that were inserted in the connection and moved vertically by 5 mm (Fig. 3c).

In the crimping tests, fixed elements were introduced to prevent the escape of the stents. Moreover, the experiments were repeated with the empty machine to assess the contribution of the friction among the crimping planes.

In the numerical model, ten rigid surfaces were introduced and radially moved to mimic the crimping testing machine. Moreover, two planes for the ID Branch and one for the ID Cav were added to prevent the stent escape, resembling the experimental set-up. To replicate the tensile test constrains, link connections between the extremities of the ID Branch and two reference points initially 6 mm spaced were used, while two semi-cylindrical rigid surfaces were defined to simulate the extension of the diaphragm (Fig. 3c).

#### Assembled System Behavior

The assembly was investigated through an axial extraction test. More specifically, the extremities of the ID Cav were fixed on a dedicated support connected to the lower clamp of the MTS machine, so that the diaphragm was centred on the testing machine axis, while the distal end of the ID Branch was blocked on a cylindrical structure fixed to the upper grip (Fig. 3b). After connecting the two components (experimentally performed by compressing the protrusion in order to elongate the proximal part of the device, introducing the flower into the diaphragm), the superior clamp was moved until the separation of the components occurred.

Numerically, three cylindrical structures were introduced and rigidly constrained to the extremities highlighted in red in Fig. 3b. First, the ID Branch proximal end was moved under force control, until the diaphragm was in line with the restriction between the flower and the protrusion (Step 1 assembly). Then, the contact among the components was activated, the load was gradually removed, and the distal end of the braided stent was concurrently approached (Step 2 assembly). Finally, the experimental extraction test was replicated by imposing a displacement of 35 mm to the distal end of the ID Branch.

#### Release Procedure

**Figure 4:**
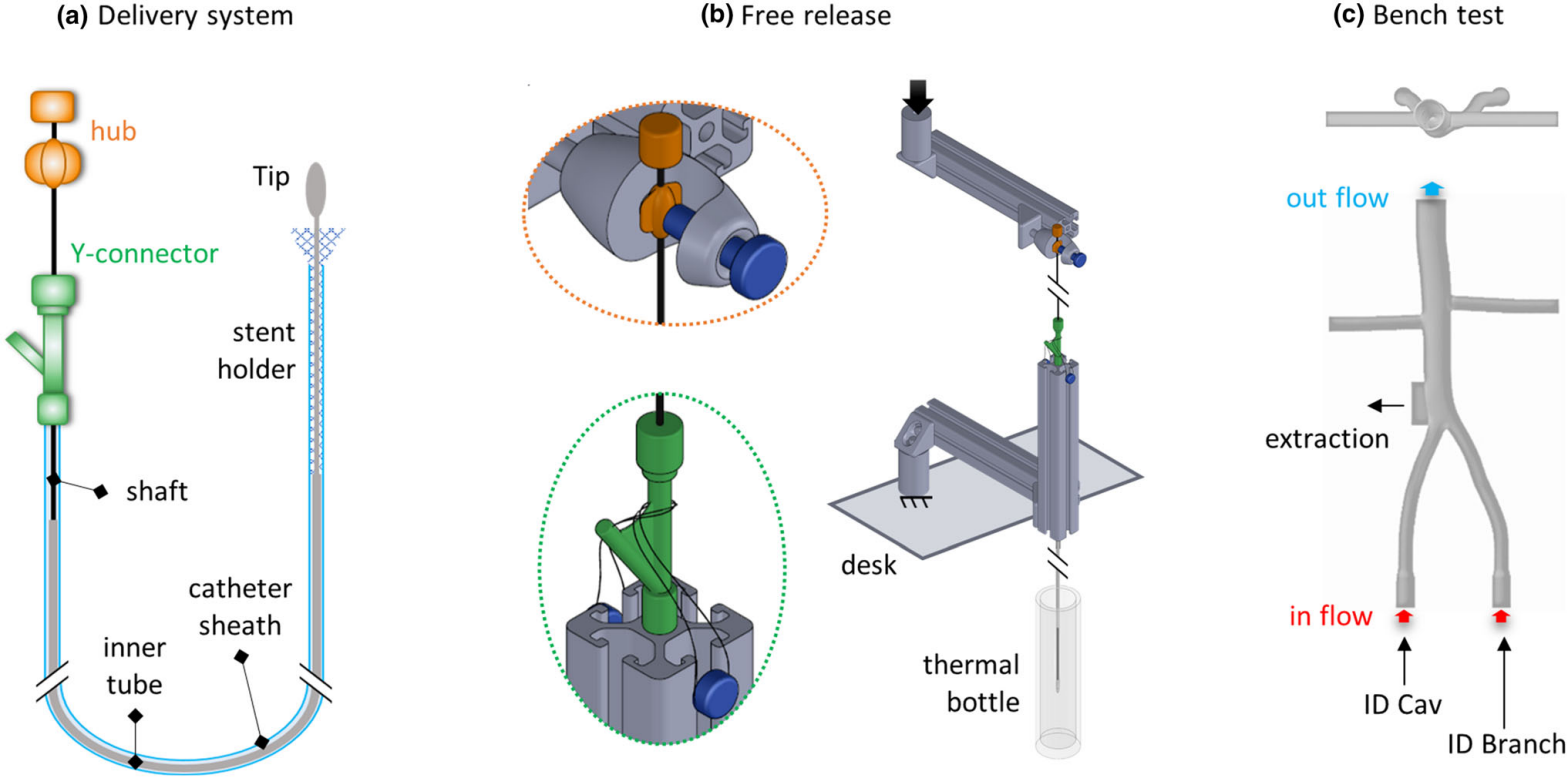
(a) Delivery system representation (colors are for illustrative purposes only). (b) Free release experimental set-up. (c) Bench test: internal flow inlet and outlet, system components’ access and extraction site.

The release procedure was investigated using free release tests, starting from the samples inserted in their dedicated delivery systems, consisting of a catheter which presents on one extremity the stent holder, delimited by a tip, and is coupled at the opposite end with a Y-connector, where the shaft is inserted (Fig. 4a). The stent holder is placed by sliding the delivery system on a guidewire previously introduced in the iliac veins. To deploy the component, the Y-connector is pulled towards the hub, held firmly in place.

The delivery system was connected to the MTS grips to measure the force required for the surgeon to deploy the device. Moreover, to perform the test with the catheter in a straight configuration, two mechanical arms were installed, placing the system beyond the testing machine desk (Fig. 4b). The hub was connected to the upper beam, and the Y-connector was fixed at the lower arm through rubber bands with adjustable tension levels, thanks to the lateral screws. The catheter was inserted in a vertical conduit to guarantee the straightness of the trait crossed by the shaft. Thus, the stent holder was placed in a thermal bottle filled with water at 37 °C (allowing deviations within ± 1 °C), to guarantee the correct temperature condition. Finally, the hub was pushed towards the fixed Y-connector, unlike during the implant procedure, resulting in the stent being released. Concerning the ID Branch, the recapture after the protrusion release, following the instruction for use, was investigated.

With regards to the numerical model, in the attempt to replicate the real insertion procedure, the ID Branch was pulled inside a rigid tube (3 mm diameter), through a funnel shape defined by a conicity of 0.32. Concerning the ID Cav, the crimping procedure was approximated by surrounding the stent with a cylinder, whose diameter was decreased to the inner catheter dimension (4 mm). Given the specific shape of the ID Cav, whose proximal extremity features a significantly higher diameter than the distal end, the crimping process was subdivided into two steps, the first of which was applied to equalize the stent dimension by crimping the proximal end. The friction coefficient, defining the interaction between the stent and the catheter, was estimated based on the release and recapture test of the ID Branch protrusion (Online Appendix C).

### Deployment in a Realistic Geometry

Finally, the deployment in a realistic geometry was performed both experimentally and numerically. An ad hoc bench test made of silicone (Fig. 4c) was immersed in a bath at 37 °C and connected with a pump and a heat exchanger to ensure an inner flow at the same temperature, allowing deviations of ± 1 °C. The implant was manually performed, inserting the delivery systems through two valves at the iliac veins inlet. First, the ID Cav was deployed, followed by the placement of the ID Branch. Finally, the system was removed from the bench test through a lateral aperture, which was closed during the procedure with a clamp.

The numerical simulation can be subdivided into 4 main phases, outlined below (see Online Appendix B for material and section details).

**Figure 5:**
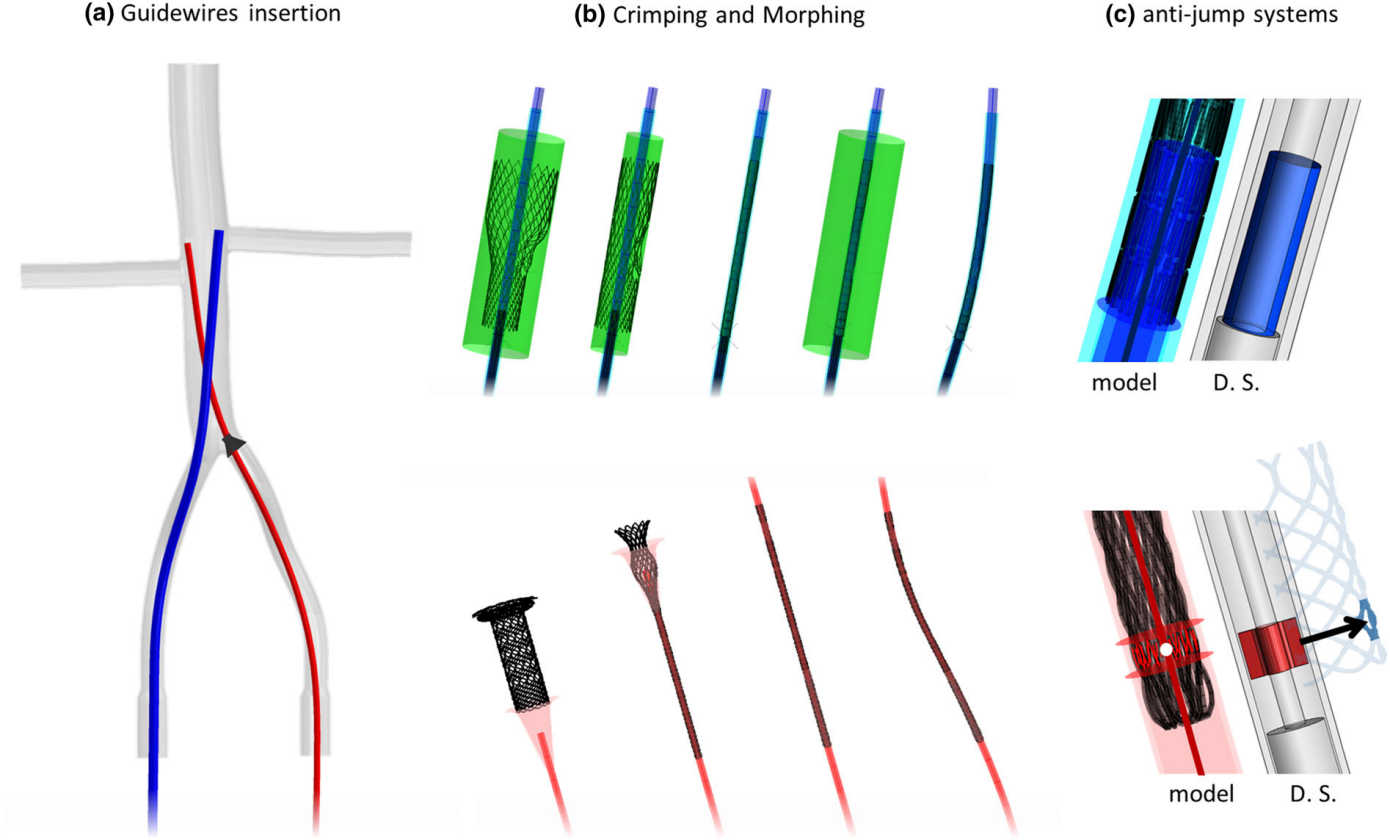
(a) Guidewires insertion: ID Cav guidewire in blue, ID Branch guidewire in red, and the funnel shape to ensure the passage through the diaphragm in black. (b) Stents insertion in the catheters through a sliding step for the ID Branch and a crimping step with the surface represented in green for the ID Cav. The deformable catheters are then morphed to conform with the guidewires’ configurations. (c) anti-jump elements in the model and illustration of the real delivery systems (D. S.). Colors are for illustrative purposes only.
- Crimping (Fig. 5b): the crimping process is outlined in the release procedure paragraph. In this test, the catheters were considered deformable.
- Morphing (Fig. 5b): for each catheter, a central wire was introduced with a diameter 0.1 mm smaller than the inner diameter of the catheter. The central wire was defined by the same number of nodes as the respective guidewire, and similarly numbered from the distal end to the proximal end. Thus, each node was moved to assume the shape of the correspondent node in the guidewire previously inserted in the bench test, deforming both the catheter, and corresponding stent, accordingly.
- Release: the catheters were then shifted, following the path defined by the central wires, gradually releasing the ID Cav followed by the ID Branch. In this step, the silicon vessel was considered deformable and the friction coefficient between the device and the bench test was set equal to 0.2^5^.

- Guidewire insertion (Fig. 5a): the bench test was fixed while two 35 cm long wires were introduced from the iliac veins’ inlet, resembling the placement of the guidewires. An ad hoc funnel shape was introduced to guarantee that the ID Branch wire passed through the Diaphragm. This simulation identified the target lines used in the following morphing phase. To prevent interpenetration between the catheters and the bench test wall in the subsequent steps, the guidewires contact diameter was increased to 5.3 mm and 3.5 mm for the ID Cav and ID Branch, respectively.

To prevent the escape of the stents, the delivery systems involve specific elements, referred to as anti-jump systems (Fig. 5c), that constrain the distal portions of the components. An additional internal tube (3 mm external diameter) compresses the last 10 mm of the ID Cav distal portion, so that the friction prevents unintended outward movements. While for the ID Branch, two winglets, connected with the inner tube, are inserted in line with the two opposite cells in the second cell line of the braided stent (Fig. 5c) when the stent is crimped on the delivery system.

The anti-jump systems were numerically represented. For the ID Cav, a rough interaction with an internal cylinder was defined. For the ID Branch, two transversal surfaces were introduced, and the contact between these surfaces and the second cell line of the stent (highlighted in Fig. 5c) was activated. The anti-jump elements should adjust their position during the morphing phase. Thus, the internal tube of the ID Cav was considered deformable, with the same properties of the external catheter, and its distal end was constrained with the closest node of the central wire. Similarly, the transversal surfaces of the ID Branch were rigidly connected with the nearest node of the deforming beam (white dot in Fig. 5c).

## Results

### Finite Element Model Validation

In the following, the mean experimental data are illustrated, while the curves for each specimen are reported in Online Appendix D.

#### Individual Components Behavior

**Figure 6:**
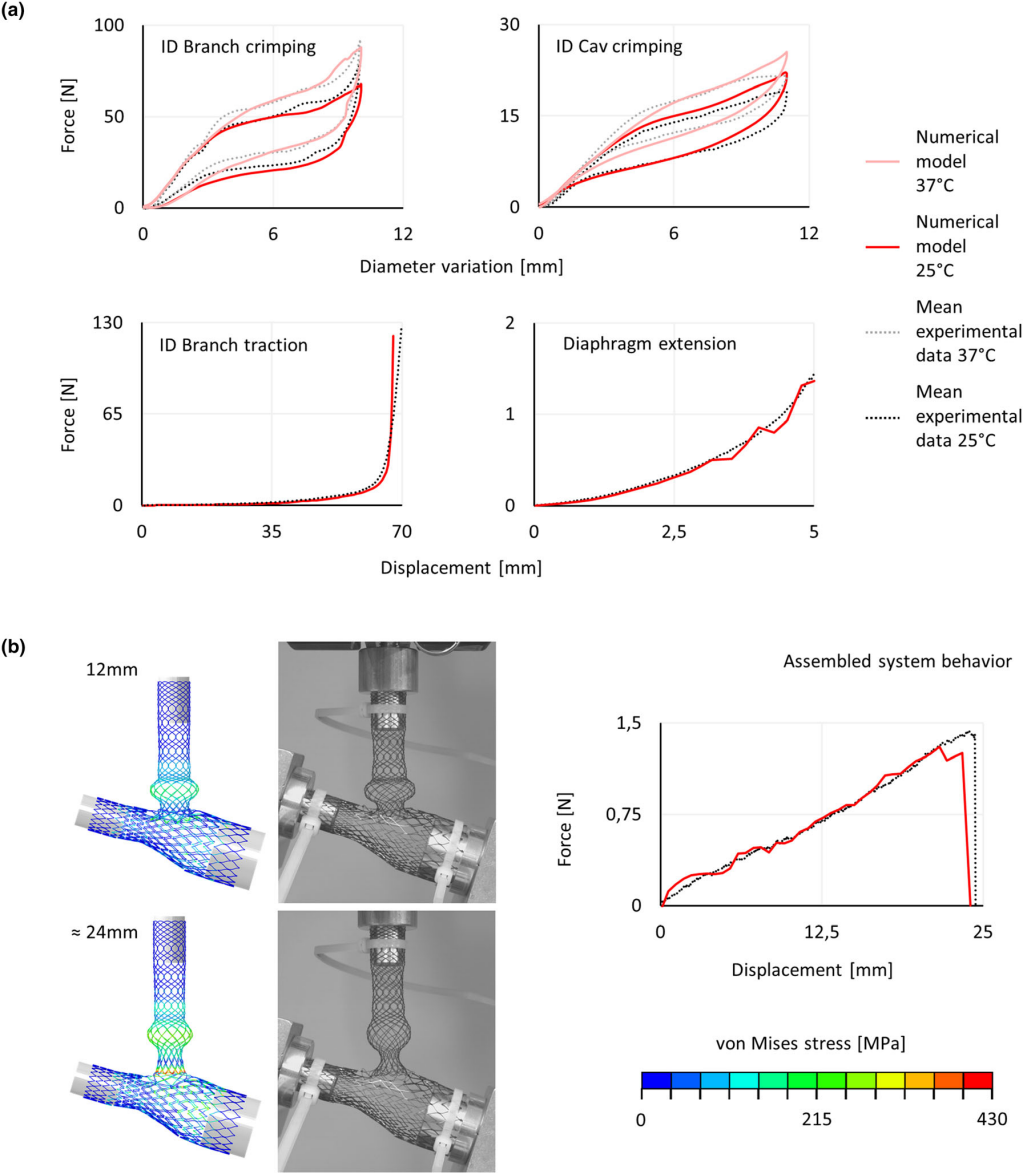
(a) Individual components behavior, comparison between experimental and numerical force-displacement curves for the: ID Branch and ID Cav crimping (at 25 and 37 °C), Diaphragm extension, and ID Branch traction. (b) Assembled system behavior, comparison between experimental and numerical results based on force-displacement curves and deformed configurations (before the separation and at about half of the maximum displacement) with the von Mises stress colored map.

Figure 6a shows the force-displacement plots. The experimental curves represent the mean of the tests performed on three different samples. The repeatability is demonstrated by the small standard deviations (maximum values below 10.5% of the maximum force in each test).

Concerning the crimping tests, the contribution of the interaction among the crimper planes was removed, as explained in the previous section. However, the friction between the stent and the testing machine strongly affects the results. Thus, the test on the ID Branch at 25 °C was used to calibrate this parameter (Online Appendix C), and the selected value, 0.05, was kept constant in the remaining three crimping tests. The models correctly predict the initial slope: 13.94 Nmm^−1^ in the range 0-2 mm for the ID Branch, and 3.32 Nmm^−1^ within 0-3 mm for the ID Cav, with an error of 6.50 and 6.41% respectively. Moreover, the slope of the plateau and its dependence on temperature variations were accurately replicated: 2.11/4.47 Nmm^−1^ at 25/37 °C in the range 5–8 mm for the ID Branch, and 1.04/1.30 Nmm^−1^ at 25/37 °C within 6–8 mm for the ID Cav, exhibiting a maximum deviation of 6.11% from the experimental data. Globally, the differences between the test results and the models remained below 10% of the maximum experimental force, except for diameter variation higher than 9 mm, where they are below 15%.

Regarding the tensile tests, the differences are lower than 8 and 11% of the maximum force value, with an average of 3.36 and 4.47%, for the diaphragm extension and for the ID Branch traction respectively, (considering displacements up to 60 mm). For the latter, above 60 mm, the errors rapidly grow due to the concavity of different curves.

#### Assembled System Behavior

Since both the system components are involved in this test, the experiment was repeated for the same ID Cav varying three different ID Branch samples and for the same ID Branch with three different ID Cav samples. The standard deviation among the experimental data does not exceed the 8.20% of the maximum force, with a mean value equal to 4.42%.

Considering displacements lower than 21.6 mm, the slope of the linear trend line is equal to 0.0579 Nmm^−1^ for the experimental data and 0.0592 Nmm^−1^ for the numerical data. This corresponds to a difference of 2.25%. For higher displacements, the model shows a drop in the force which leads to a difference in the load peak value of 11.89% (1.26 N in contrast to 1.43 N). Nevertheless, the separation of the components was registered at 24.00 mm, remaining within the experimental interval of uncertainty (24.40 ± 1.54 mm). Figure 6b reports the force-displacement plots and the system configurations before the separation (≈ 24 mm) and at roughly half of the maximum displacement (12 mm). In both instants, the deformations are coherent with the experimental observations, except for the reduced restriction between the flower and protrusion traits at displacements near the separation extension.

#### Release Procedure

**Figure 7:**
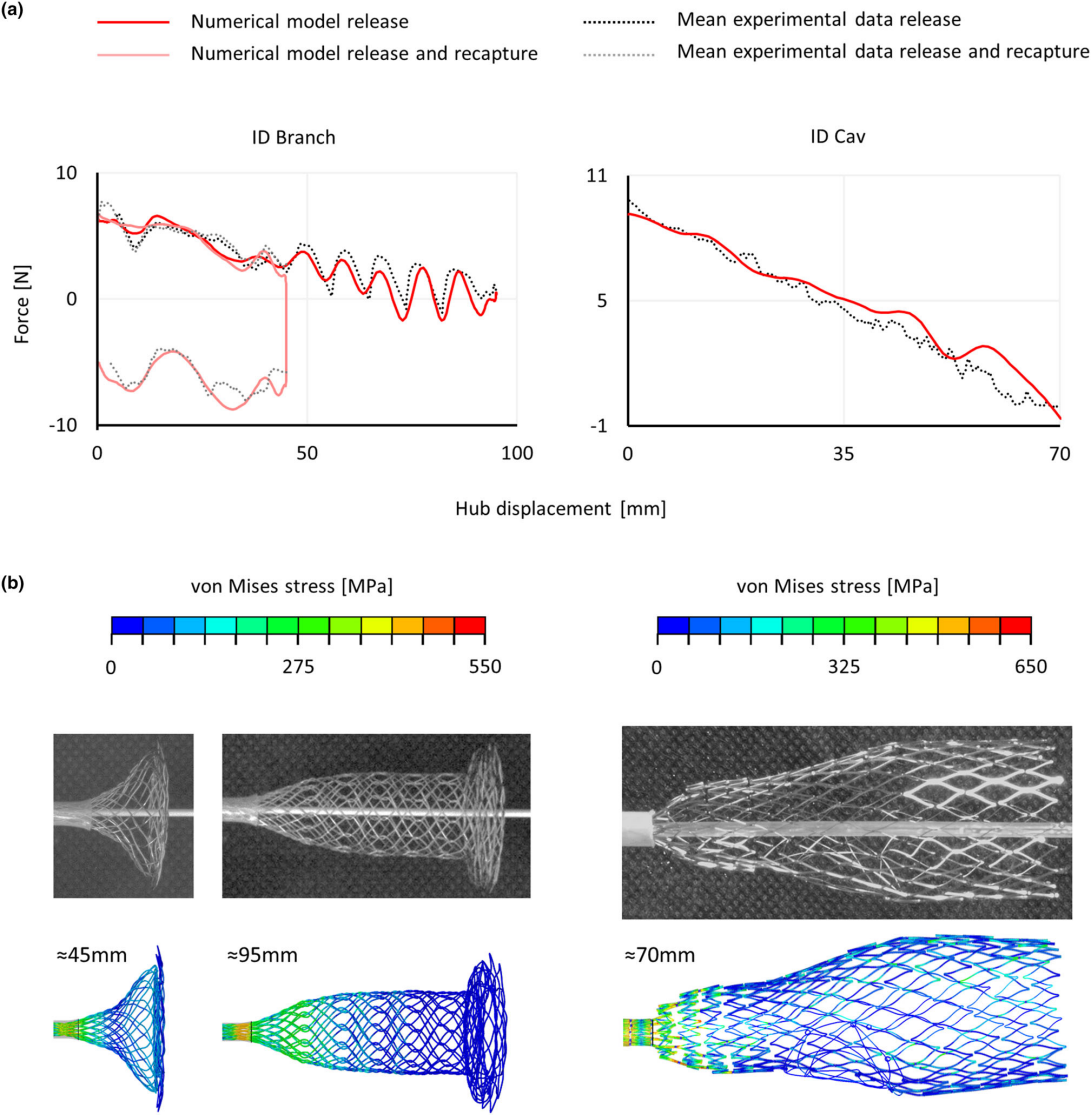
Free release tests: comparison between experimental and numerical force-displacement curves (a), and deformed configurations with the von Mises stress colored map (b).

The force measured by the MTS machine during the free release tests exhibits a descending trend and, considering the ID Branch, it presents some distinctive oscillations because of the flower (≈ 9.1 mm), protrusion (≈ 37.6, ≈ 45.2 mm) and double twisted traits release (≈ 55.8, ≈ 64.6, ≈ 73.5, ≈ 82.1, ≈ 91.6 mm) Fig. 7a). The mean standard deviation is equal to 0.212 N for the ID Branch and 0.589 N for the ID Cav, both below 6% of the maximum force. However, concerning the ID Branch, the standard deviation exceeds the 20% for the drops in force related to the double twisted traits release. Based on the force recorded once the stent was completely released, the delivery system contribution was subtracted. Moreover, given the articulated experimental set-up, some spurious movements arise, especially regarding the grips where the force transmitted through the extension apparatus presents a higher lever arm. Focusing on small displacements (< 10 mm), a linear increase of the load, associated with a delay in the stent releasing, was observed and connected with the deformation of the experimental set-up. Thus, the mean slope among these traits was calculated (K_set-up_^ID Branch^ = (0.93 ± 0.02) Nmm^−1^, K_set-up_^ID Cav^ = (1.61 ± 0.23) Nmm^−1^), and the displacement was adjusted based on the following equation in which F is the force and Disp_0_ is the original displacement.

$$Disp = Disp_{0} - F/K_{set - up}$$

Concerning the numerical simulations, a considerable noise was recorded. Consequently, a Butterworth filter was applied to obtain the analysed data (Online Appendix D). The friction coefficient was set equal to 0.03, based on the release and recapture test of the ID Branch (Online Appendix C). Indeed, with this value, the mean and maximum difference between the release and recapture curves are equal to 11.10 N and 15.48 N, corresponding to a difference of 1.22 and 1.53% with respect to the experimental curves. The simulations also correctly reflect the release tests (Fig. 7a) with differences less than 15%, with the exception of the ID Branch in the proximity of the double twisted traits release. Moreover, the deformed configurations are consistent with the experiments (Fig. 7b).

### Deployment in a Realistic Geometry

**Figure 8:**
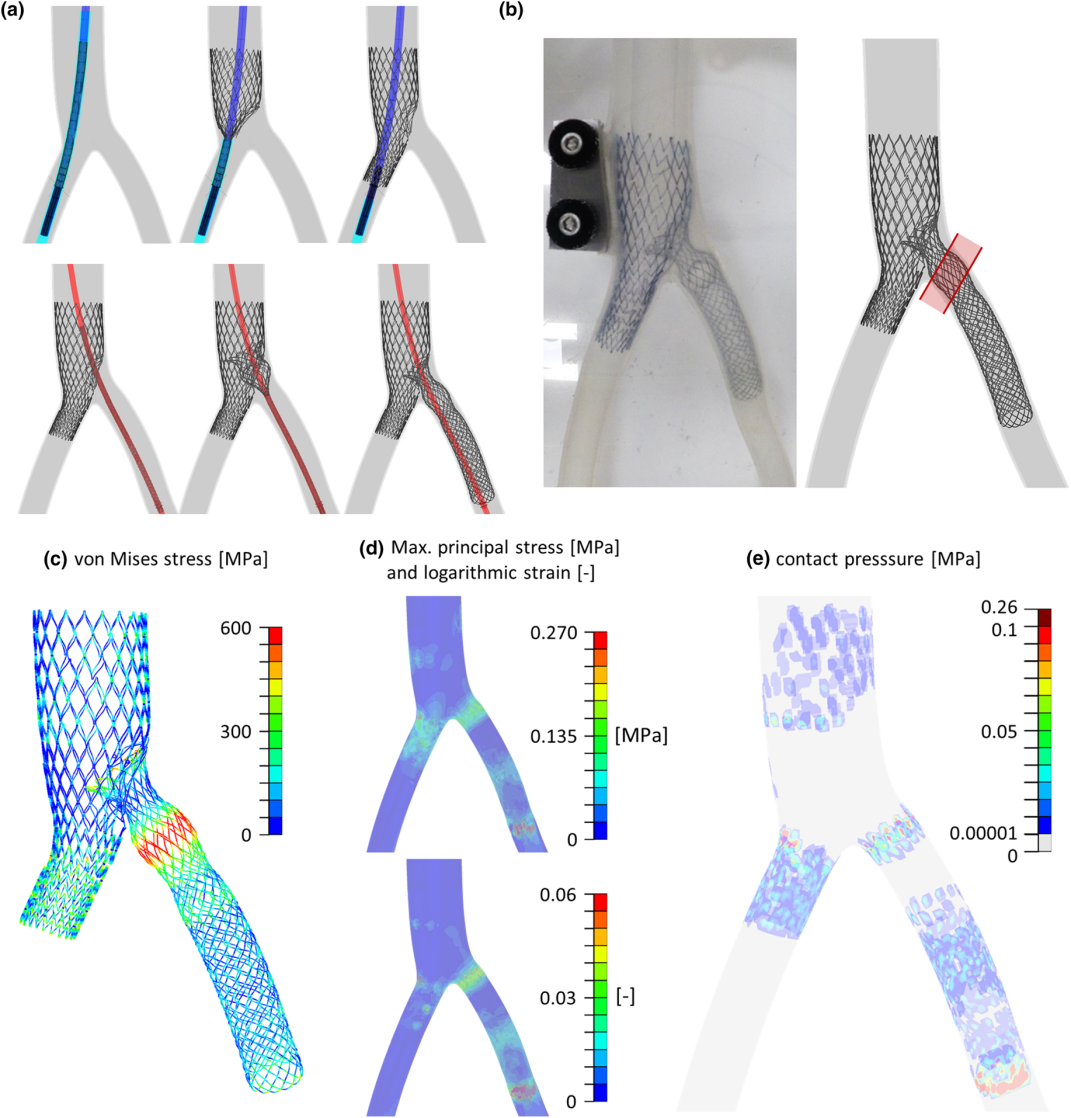
Deployment in a realistic geometry: (a) numerical deployment procedure; (b) comparison between experimental and numerical deformed configurations (the approximate region compressed by the RCIA is highlighted in red); (c) ID Venous System von Mises stress [MPa] colored map; (d) bench test maximum principal stress [MPa] and logarithmic strain [−] colored map; (e) bench test contact pressure [MPa] colored map.

The deployment procedure is illustrated in Fig. 8a. First, the ID Cav was released, placing the fenestration directed towards the LCIV inlet. This was followed by, the ID Branch being deployed so that the diaphragm lay between the flower and the protrusion trait. The final deformed configuration correctly reflects the experimental observations (Fig. 8b). The ID Branch protrusion extends to the pathological region (highlighted in red in Fig. 8b) and records the highest stress values, close to 600 MPa (Fig. 8c). Concerning the contact pressure on the wall (Fig. 8e), the highest values correspond with the ID Branch distal segment and in the proximity of the bifurcation (0.26 MPa).

## Discussion

The ID Venous System can provide a viable alternative to locally treat the May-Thurner syndrome, limiting the impact on close and healthy tissues. However, preliminary trials are needed to ensure the safety and efficacy of the device, included computational simulations. Finite element models are powerful tools that allow the prediction of device behavior subject to complex external loads, providing information on local quantities (strains and stresses) not measurable via other means. Nevertheless, given the impact on the decision process, model validation is essential. In this work, the model implementation is outlined, and illustrated following a step-by-step validation process to evaluate potential sources of uncertainty.

The model implementation required the definition of:

- Geometry: stent pictures and the drawings used during the laser-cut procedure were exploited to reconstruct the 3D models of the system components. Attention was given to measure the dimensions of the struts on samples after they underwent electropolishing.
- Mesh: beam elements were selected to minimize the computational cost.
- Material properties: axial tensile tests on wires and multi-wire samples were used to identify the constants defining the NITI superelasticity at both 25 °C and 37 °C, assuming symmetry between tensional and compressional deformations.
- Interaction properties: The general contact algorithm was exploited, specifying three different friction coefficients, describing: the contact within the stent components, the contact between the device and the bench test, and the contact between the device and the delivery systems. The first two contacts were defined based on data found in literature, while the friction with the delivery systems, given the high impact on the release and recapture force, was calibrated and subsequently confirmed based on the comparison with the free release tests.

To validate the defined model, numerical and experimental results were compared in terms of reaction forces and deformed configurations, following a sequence of tests of increasing complexity.

- Individual components behavior: both the geometry and the material approximations may affect the simulations. Thus, a single validation experiment is not sufficient to prove the validity of the stent models as some errors can compensate each other. For this reason, two tests for each component were carried out. Moreover, the tests were selected to evaluate functional features, namely, the radial stiffness and the interaction between the components. All the evidence is in agreement with the experimental results. The only exception resides in the ID Branch axial stiffness at high deformations. However, the maximum elongation reached by the stent during the implant procedure is equal to 59.5 mm (crimped configuration inside the catheter), which is within the trustworthy displacement interval. In the diaphragm extension test (Fig. 6a), the oscillations above 2.5 mm, are probably due to internal contact among the wires that are progressively compressed together.
- Assembled system behavior: the connection is a key aspect to be assessed since its functioning prevents stent migration and affects the system flexibility. Both the stiffness and maximum elongation were evaluated. The experimental and numerical results are very similar, differing only for the flower configuration. This is probably due to the geometry reconstruction procedure. Indeed, the exact process followed during manufacturing is unknown. Thus, even if the design flow illustrated in this study provided satisfactory results, some errors might derive from this missing information.
- Free release: the trend of the force-displacement plots is coherent with the experimental observation for both the ID Cav and ID Branch components. Based on the numerical simulations, the force required to deploy both the ID Cav and ID Branch, and to recapture the ID Branch lies below 10, 7 and 9 N, respectively. Note that the experimental data was preliminarily processed to remove the contribution of the set-up and the empty delivery systems. If the original mean experimental data is considered, the forces require an increase of about 0.5 N for the ID Branch and almost 1.5 N for the ID Cav. The foreshortening is a crucial feature in braided stent deployment, hindering the positioning process. The correct relationship between radial and longitudinal deformations in the model was demonstrated by the length of the ID Branch crimped inside the catheter, 102.51 mm only 1.3% longer than the experimental configuration.

Finally, given the similarity between experimental and numerical results, it is possible to declare the model reliable for the intended use, in accordance with the standard ASME V&V 40^3^ with reference to the risk level 3. The low standard deviations demonstrate the experimental reliability. Concerning the model verification, the mesh sensitivity and the estimated discretization error (Online Appendix A) prove the accuracy of the adopted mesh. Moreover, as required by the standard, the differences between experimental and numerical results remain below 10% within the range of interest. The reported deviations can be as a result of the approximations connected with geometry (design and struts/wires section dimensions), materials (material parameters and superelastic model approximations (Online Appendix B)), interactions (approximated contact detection due to beam meshes, interactions description), and boundary conditions (experimental set-up/delivery systems representation).

The numerical deployment in the bench test reflects the experimental observations, with some differences mainly due to prospective effects, proving the feasibility of the ID Venous System implant and highlighting the capability of the model to predict implant outcomes in realistic geometries. Moreover, the finite element model provides additional information, such as the maximum principal stress (Fig. 8d) being less than 0.27 MPa in the wall (below the vein failure stress^12^), and less than 1000 MPa in the device, (below the yielding point, see Online Appendix B). This proves that there is no risk of rupture or plasticity during the implanting of the stents.

Nevertheless, a weakness of the present system is evident, both in the experimental and numerical tests (Fig. 8b). Namely, to exert a sufficient pressure and restore the correct flow in the iliac vein, the cylindrical portion of the ID Branch, reinforced with double twisted traits, is intended to act on the compressed vessel region (highlighted in red in Fig. 8b). However, due to the excessive diameter of the protrusion, this trait extends up to the pathological site, exposing the more compliant portion of the ID Branch to the artery compressive action, endangering the system efficacy. To redress the outlined limitation, both a design evolution to reduce the protrusion deformation and a specific delivery system to improve the deployment process are already under evaluation.

Future developments should involve the model update with the improved design and the analysis of the system response to external loads, simulating the effect of the surrounding tissues like the cyclic load exercised by the RCIA branch. Moreover, the validated model could be used to predict the implant outcomes in patient specific anatomies. Finally, it could be useful to extend this short-term study to assess long-term issues like abrasion and fatigue failure, exploiting 3D elements to better investigate contact areas.

## Electronic supplementary material

Below is the link to the electronic supplementary material.

Supplementary material 1 (PDF 1904 kb)

## References

[1] Anderson AE, Ellis BJ, Weiss JA (2007). Verification, validation and sensitivity studies in computational biomechanics. Comput. Methods Biomech. Biomed. Engin..

[2] ASME. V&V 10: Guide for verification and validation in computational solid mechanics., 2006.

[3] ASME. V&V 40: Assessing credibility of computational modeling through verification and validation: application to medical devices., 2018.

[4] Batagini NC, Hardy D, Clair DG, Kirksey L (2016). Nellix endovascular Aneurysm Sealing System: device description, technique of implantation, and literature review. Semin. Vasc. Surg..

[5] De Bock S, Iannaccone F, De Santis G, De Beule M, Van Loo D, Devos D, Vermassen F, Segers P, Verhegghe B (2012). Virtual evaluation of stent graft deployment: A validated modeling and simulation study. J. Mech. Behav. Biomed. Mater..

[6] Chakfe N, Nicolini P, Contassot D (2020). Flat spring to ensure an elastic and compliant branch connection between two stents. Eur. J. Vasc. Endovasc. Surg..

[7] Date Y, Takano T, Fujii T, Terasaki T, Sakaguchi M (2019). Double D technique: an innovative modified bifurcated stent graft deployment strategy for an isolated common iliac artery aneurysm with a challenging renal artery anatomy. Vasc. Endovascular Surg..

[8] Derycke L, Perrin D, Cochennec F, Albertini JN, Avril S (2019). Predictive Numerical Simulations of Double Branch Stent-Graft Deployment in an Aortic Arch Aneurysm. Ann. Biomed. Eng..

[9] Erdemir A, Guess TM, Halloran J, Tadepalli SC, Morrison TM (2012). Considerations for reporting finite element analysis studies in biomechanics. J. Biomech..

[10] Finazzi V, Demir AG, Biffi CA, Chiastra C, Migliavacca F, Petrini L, Previtali B (2019). Design rules for producing cardiovascular stents by selective laser melting: Geometrical constraints and opportunities. Procedia Struct. Integr..

[11] Hall GJ, Kasper EP (2006). Comparison of element technologies for modeling stent expansion. J. Biomech. Eng..

[12] Hamedani BA, Navidbakhsh M, Tafti HA (2012). Comparison between mechanical properties of human saphenous vein and umbilical vein. Biomed. Eng. Online.

[13] Henninger HB, Reese SP, Anderson AE, Weiss JA (2010). Validation of computational models in biomechanics. Proc. Inst. Mech. Eng. Part H..

[14] Hulsberg PC, McLoney E, Partovi S, Davidson JC, Patel IJ (2016). Minimally invasive treatments for venous compression syndromes. Cardiovasc. Diagn. Ther..

[15] Karanasiou GS, Papafaklis MI, Conway C, Michalis LK, Tzafriri R, Edelman ER, Fotiadis DI (2017). Stents: biomechanics, biomaterials, and insights from computational modeling. Ann. Biomed. Eng..

[16] Kelly N, McGrath DJ, Sweeney CA, Kurtenbach K, Grogan JA, Jockenhoevel S, O’Brien BJ, Bruzzi M, McHugh PE (2019). Comparison of computational modelling techniques for braided stent analysis. Comput. Methods Biomech. Biomed. Engin..

[17] Knuttinen MG, Naidu S, Oklu R, Kriegshauser S, Eversman W, Rotellini L, Thorpe PE (2017). May-Thurner: diagnosis and endovascular management. Cardiovasc. Diagn. Ther..

[18] Ligi D, Croce L, Mannello F (2018). Chronic venous disorders: the dangerous, the good, and the diverse. Int. J. Mol. Sci..

[19] Morlacchi S, Migliavacca F (2013). Modeling stented coronary arteries: where we are, where to go. Ann. Biomed. Eng..

[20] Morris PD, Iqbal J, Chiastra C, Wu W, Migliavacca F, Gunn JP (2018). Simultaneous kissing stents to treat unprotected left main stem coronary artery bifurcation disease; stent expansion, vessel injury, hemodynamics, tissue healing, restenosis, and repeat revascularization. Catheter. Cardiovasc. Interv..

[21] Murphy E (2019). Surveying the 2019 venous stent landscape. Endovasc. Today.

[22] Radaideh Q, Patel NM, Shammas NW (2019). Iliac vein compression: epidemiology, diagnosis and treatment. Vasc. Health Risk Manag..

[23] Sargent RG (2013). Verification and validation of simulation models. J. Simul..

[24] Shabani Varaki E, Gargiulo GD, Penkala S, Breen PP (2018). Peripheral vascular disease assessment in the lower limb: a review of current and emerging non-invasive diagnostic methods. Biomed. Eng. Online.

[25] Verheye, S., S. Ramcharitar, E. Grube, J. J. Schofer, B. Witzenbichler, J. Kovac, K. E. Hauptmann, P. Agostoni, M. Wiemer, T. Lefèrév, R. Spaargaren, P. W. Serruys, H. M. García-García, and R. J. Van Geuns. Six-month clinical and angiographic results of the STENTYS® self-apposing stent in bifurcation lesions. *EuroIntervention* 7:580–587, 2011. 10.4244/EIJV7I5A94 21930462

[26] White JM, Comerota AJ (2017). Venous Compression Syndromes. Vasc. Endovascular Surg..

[27] Zaccaria A, Migliavacca F, Pennati G, Petrini L (2020). Modeling of braided stents: Comparison of geometry reconstruction and contact strategies. J. Biomech..

