# Supplementary material for: Finite Element Simulations of the ID Venous System to Treat Venous Compression Disorders: From Model Validation to Realistic Implant Prediction
Source: Ann Biomed Eng. 2021 Jan 4;49(6):1493–506. doi: 10.1007/s10439-020-02694-8 (PMC8137589; doi:10.1007/s10439-020-02694-8)
Supplement: Supplementary file 1 — Supplementary material 1 (PDF 1904 kb) [file 10439_2020_2694_MOESM1_ESM.pdf]

Appendix A: Mesh sensitivity

The mesh sensitivity was carried out on single units of the device descriptive of the ID Branch, ID Cav, and Diaphragm components. All the units were meshed with both 3D (C3D8I) and beam elements (B31) to select the best compromise between computational cost and model accuracy. A MATLAB code was implemented to assign the correct beam orientation[2].

One extremity was kept fixed, while a linear displacement of 15 mm, 1.5 mm, and 2.5 mm for the ID Branch, ID Cav, and Diaphragm units, respectively, was applied at the opposite end (Figure 1A).

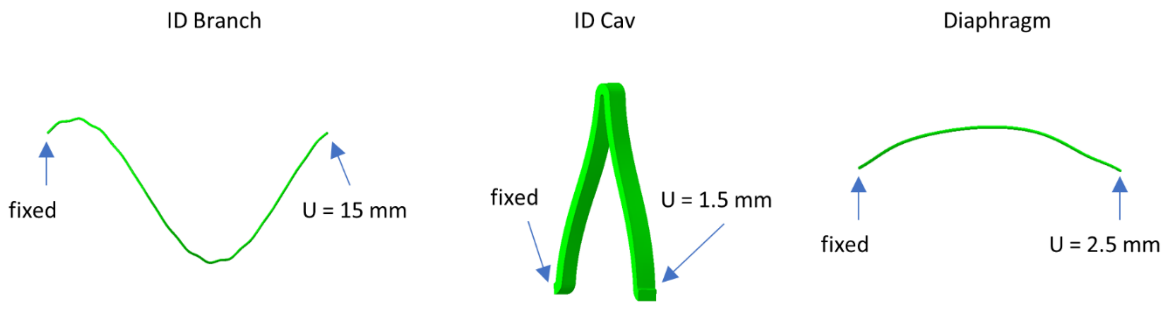

FIGURE 1A. Units of the device with the applied boundary conditions.

3D and beam mesh were compared in terms of reaction force, maximum von Mises stress, and computational time (Table 1A).

Concerning the 3D elements, the differences between the medium and fine mesh are lower than 10%, with a computational time saving of approximately 50%.

Regarding the beam elements, the differences between the coarse and fine mesh are lower than 2.0%, 8.0%, 1.4% for the ID Branch, ID Cav, and diaphragm, respectively. However, the gain in computational time is not significant with respect to the medium mesh, and given the better curvature approximation, the medium mesh was selected. The B31 fine mesh was discarded to minimize the reduction of the contact thickness in the general contact algorithm.

TABLE 2A. Comparison among 3D (C3D8I) and beam (B31) meshes: number of elements (#el); percentage difference with respect to the corresponding fine mesh in terms of von Mises stress (ΔSM%) and reaction force (ΔRF%); percentage of computational time saved (Δtime%). On the bottom, the differences of the B31 fine mesh with respect to (wrt) the 3D fine mesh are reported.

	ID Branch				ID Cav				Diaphragm			
					**C3D8I**							
	# el	ΔSM%	ΔRF%	Δtime%	# el	ΔSM%	ΔRF%	Δtime%	# el	ΔSM%	ΔRF%	Δtime%
Coarse	6240	2	10	83	1512	12	3	80	4764	6	13	81
Medium	18018	1	3	50	3648	8	2	49	12516	1	7	51
Fine	36608				7455				25408			
					**B31**							
	# el	ΔSM%	ΔRF%	Δtime%	# el	ΔSM%	ΔRF%	Δtime%	# el	ΔSM%	ΔRF%	Δtime%
Coarse	52	2	0.5	29	12	8	6	11	25	0.4	1.4	26
Medium	104	0.5	0.2	21	24	7	4	8	51	0.2	0.6	21
Fine	208				48				100			
B31 Fine wrt C3D8I Fine		6	5	99.9		2	2	99.7		6	1.4	99.9

Given the small differences and the significantly lower computational time, the beam mesh was preferred to the 3D option. Indeed, the force-displacement trends and the deformed configurations are very similar. The comparison between the results obtained with the selected mesh (B31 medium) and the 3D finest one is reported in Figure 2a.

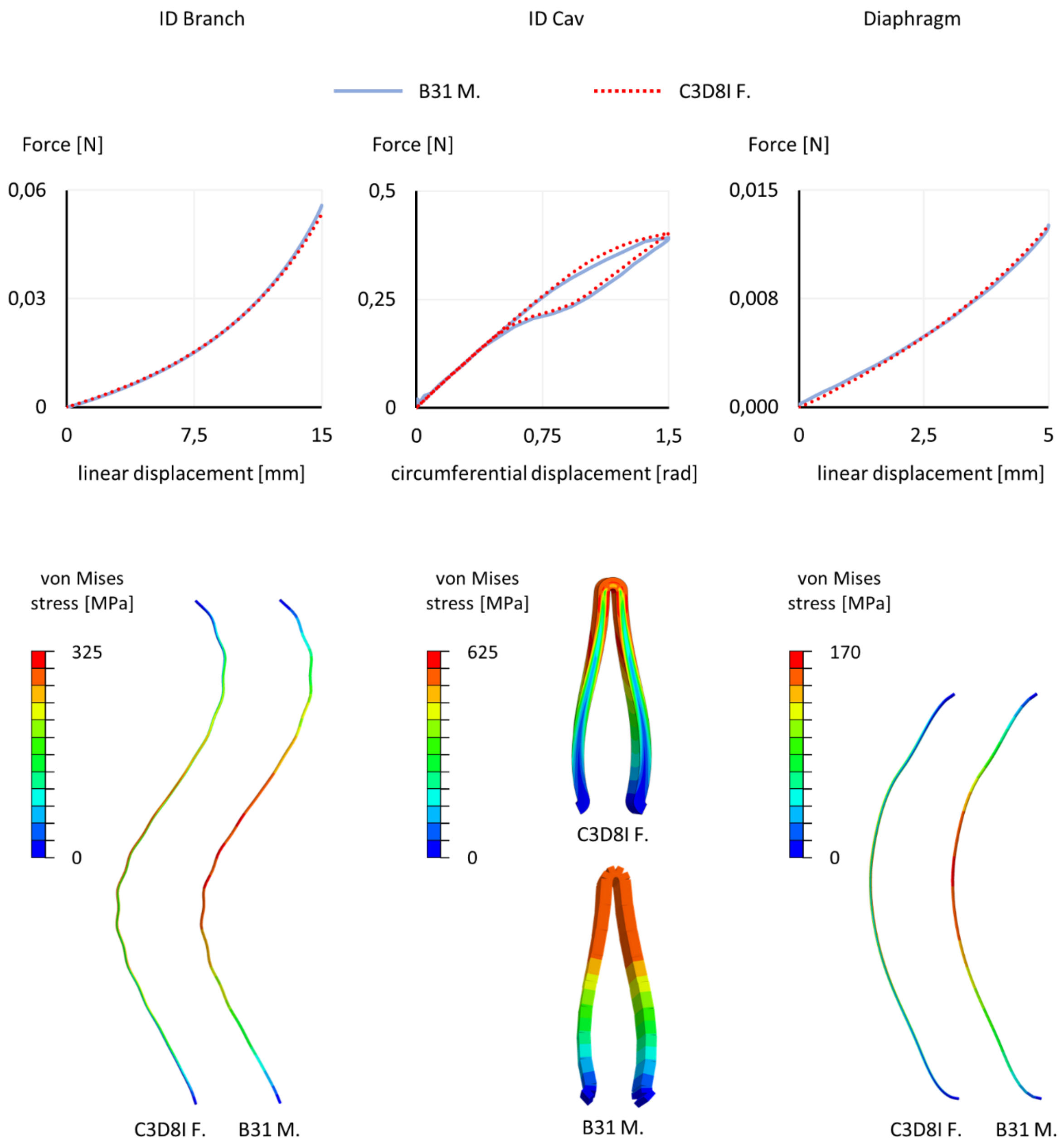

FIGURE 2A. Comparison between B31 medium mesh (B31 M.) and C3D8I fine mesh (C3D8I F.) in terms of force-displacement curve (top) and deformed configuration with von Mises stress colored map (bottom).

Appendix B: Material parameters and cross-section dimensions

1. <u>ID Venous System</u>

The ID Venous System is made of Nitinol, a Nickel-Titanium alloy featuring a superelastic behavior above a specific temperature value (T>Af). The material parameters were calibrated based on tensile tests at room and body temperature, 25°C and 37°C (both above Af) performed on wire samples, in the case of ID Branch and diaphragm, and on multi-wire specimens[1], laser-cut from the same tube used for the ID Cav with section dimensions reflecting the stent struts (Figure 1Ba). The results of tests up to fracture allowed also to identify the yielding of the material (above this value there is no more a complete recovery of the deformation during unloading and a small increment of stress induces fracture).

An MTS 858 Mini Bionix servohydraulic testing machine under displacement control (displacement range 0-10 mm, load cell 1500 N) was used for the mechanical tests. A temperature control system was set-up, consisting of a commercial hydraulic pump (Watson Marlow 520 DU), an upstream reservoir with controlled water temperature (Julabo MA Heating Immersion Circulator), and an ad-hoc plexiglass chamber (Figure 1Bb).

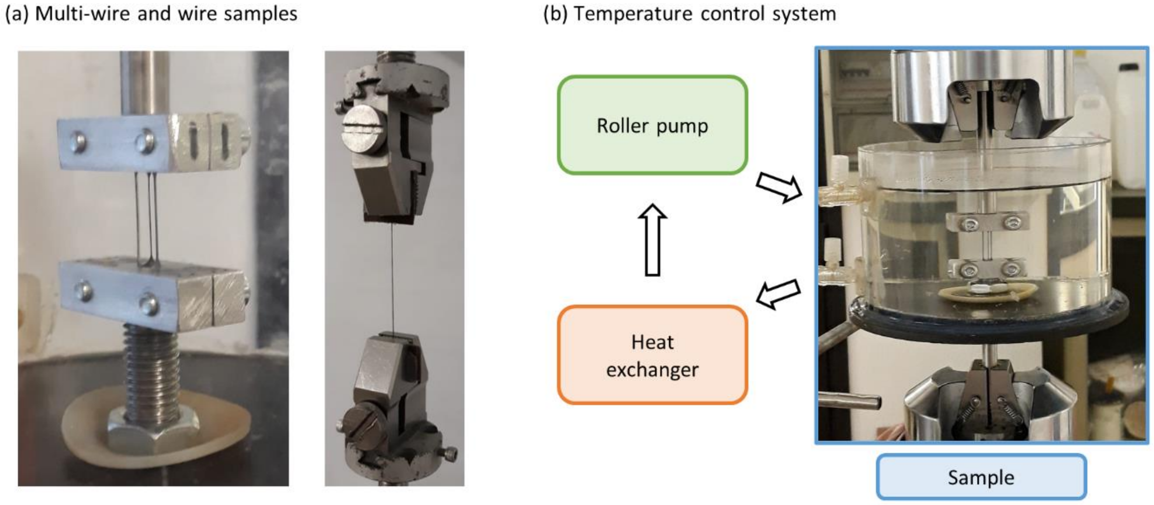

FIGURE 1B. (a) multi-wire sample (left) and wire sample (right) with related gripping system. (b) Experimental set-up and heating system for material parameters calibration.

Based on the initial length (L_0) and cross-sectional area (A_0) of the specimens, the engineering and true stress and strain values were evaluated according to the formulae:

$$\sigma_{eng} = \frac{F}{A_0} \qquad \varepsilon_{eng} = \frac{\Delta L}{L_0}$$

$$\sigma_{true} = \sigma_{eng}(1 + \varepsilon_{eng}) \qquad \varepsilon_{true} = ln(1 + \varepsilon_{eng})$$

The constitutive parameters describing the Nitinol superelastic behavior (Table 1B) were deduced from the true stress-true strain curves.

TABLE 3B. Superelastic material parameters: graphical representation and definition.

SUPERELASTIC MATERIAL PARAMETERS

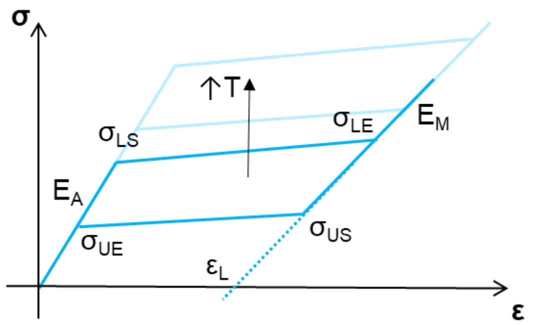

**E_A**	austenite (A) elastic modulus
**E_M**	martensite (M) elastic modulus
**ν**	Poisson's ratio (assumed equal to 0.3)
**ε_L**	transformation strain
**σ_{LS}**	Start of the transformation phase during Loading (A➔M)
**σ_{LE}**	End of transformation phase during Loading (A➔M)
**σ_{US}**	Start of transformation phase during Unloading (M➔A)
**σ_{UE}**	End of transformation phase during Unloading (M➔A)
**T_0**	reference temperature
**(δσ/δT)**	Temperature dependence of the stress values defining the transformation plateau

The selected parameters were optimized based on computational simulations. More precisely, the values were obtained simulating the uniaxial loading of an ideal cube (Figure 2Ba), (Table 2B, Figure 2Bc). Concerning multi-wire samples, a second step was performed: the real specimen geometry was reconstructed, and a repetitive unit was isolated and meshed with 6840 hexahedral elements (Figure 2Bb); then a tensile simulation replicating the experimental tests was performed by imposing a circumferential symmetry on the lateral faces; finally, the parameters were modified in order to improve the match between the model and the experimental results in terms of force-displacement curves (Table 2B, Figure 2Bd). This process allows calibrating the material parameters, accounting also for the effects connected with the dog-bone shape of the multi-wire strands. Note that some approximations of the Abaqus built-in super-elastic material model are present. In particular, the model uses the same slope for describing the martensite elastic behavior during loading (at the end of the upper transformation plateau) and during unloading (before the start of the lower transformation plateau), underestimating the experimental hysteresis (Figure 2Bc-d).

TABLE 2B. Parameters used to define the ID Branch, Diaphragm and ID Cav material behavior. The values were optimized based on numerical simulations on an ideal cubical geometry (cube), and, for the ID Cav, on realistic multi-wire geometry to account for the dog bone shape (multi-wire).

Material parameter	Units	ID Branch cube	Diaphragm cube	ID Cav cube	ID Cav multi-wire
E_A	MPa	45000	50000	60000	70000
E_M	MPa	20000	20000	18000	25000
ν	-	0.3	0.3	0.3	0.3
ε_L	-	0.05	0.045	0.0355	0.04
σt_L^S	MPa	450	430	410	420
σt_L^E	MPa	520	500	470	520
σt_U^S	MPa	230	230	180	220
σt_U^E	MPa	200	190	150	120
T_0	°C	25	25	25	25
$(\Delta\sigma/\Delta T)$	MPa/°C	11	11	11	11

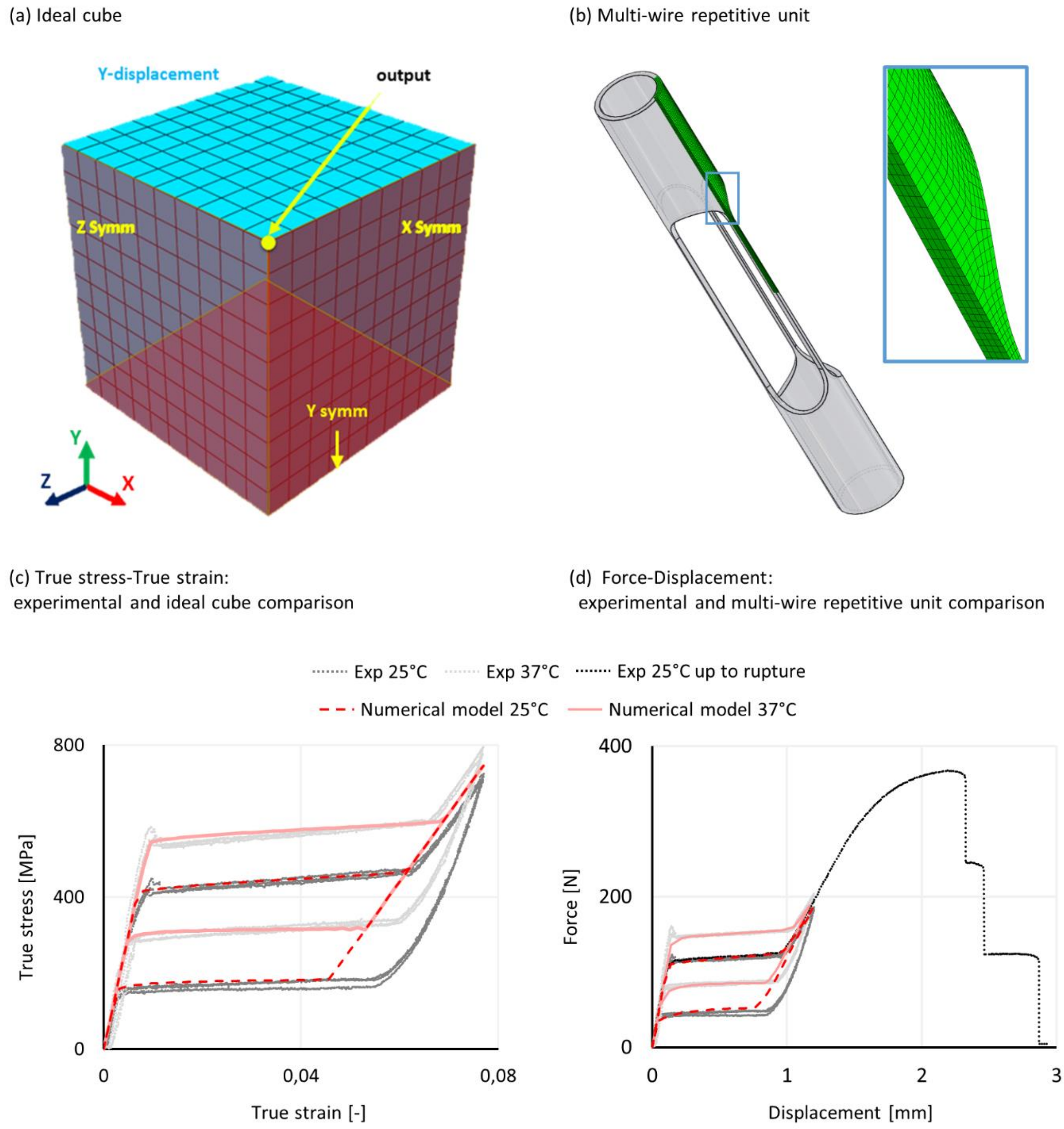

FIGURE 2B. (a) Cube simulations: model and boundary conditions. (b) Multi-wire geometry (grey) and repetitive unit (green) with mesh magnification. (c) True stress – True strain plot: comparison between experiments and ideal cube simulations at 25°C and 37°C. (d) Force – Displacement plot: comparison between experiments and multi-wire repetitive unit simulations at 25°C and 37°C.

Finally, to ensure the similarity between real devices and in-silico models, the struts and wires dimensions were defined based on average values extracted from microscopy images of stents after electropolishing, of which an example is illustrated in the Figure below.

ID Cav strut width

Mean	91,8
Dev. St.	10,1

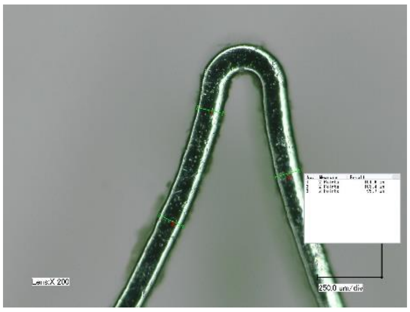

ID Cav link width

Mean	313,2
Dev. St.	7,0

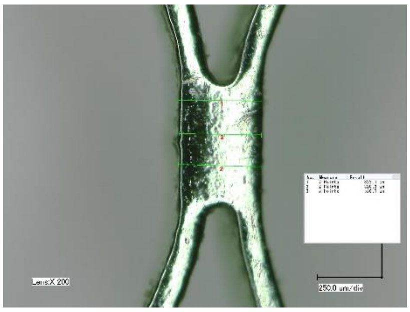

ID Cav strut/link thickness

Mean	418,4
Dev. St.	11,9

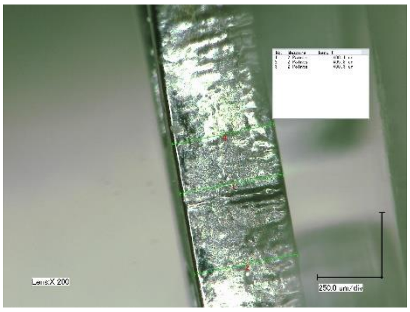

ID Branch wires diameter

Mean	221,5
Dev. St.	6,6

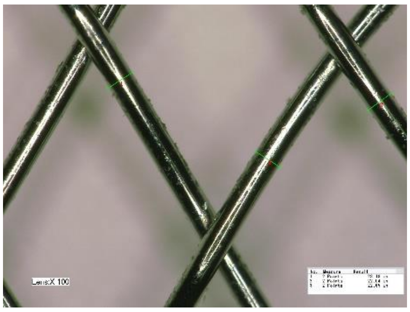

Diaphragm wire diameter

Mean	131,2
Dev. St.	4,6

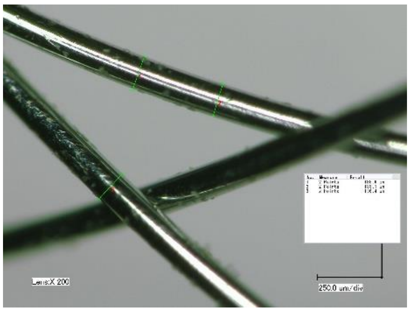

FIGURE 3B. ID Cav, ID Branch and Diaphragm beam section dimensions.

2. Bench test

The elastic modulus of the silicon vessel was calibrated based on a pressurization test. The portion resembling the LCIV was occluded at about half of its length, and fluid was injected from the vein inlet, measuring the pressure and the related external diameter variation. The elastic modulus (E), reported in Table 3B, was evaluated based on the following equations, under the thin-walled assumption:

$$\sigma = \frac{\Delta P \cdot D}{2t} \qquad \varepsilon = \frac{(D - D_0)}{D_0} \qquad E = \frac{\sigma}{\varepsilon}$$

where σ and ε are the stress and strain in the wall, ΔP is the pressure variation, D and D_0 are the final and initial diameter, and t the wall thickness (1 mm).

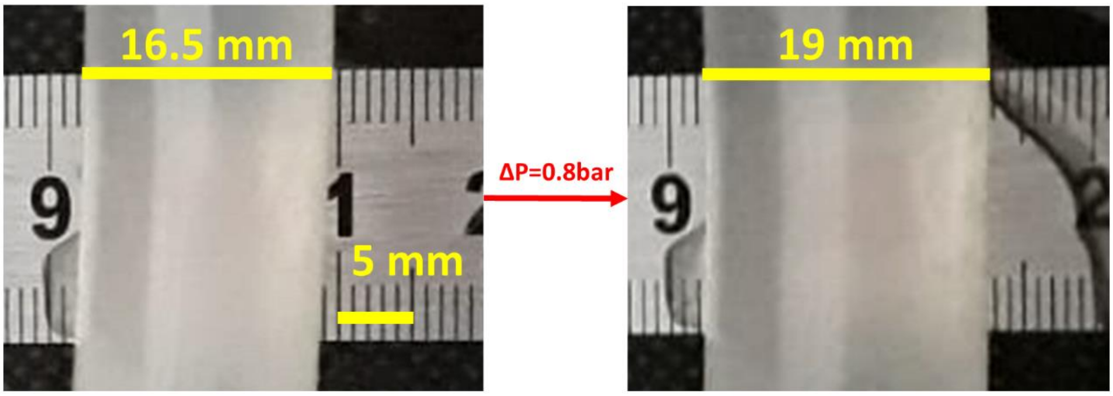

FIGURE 4B. Bench test pressurization test: diameter variation due to pressure variation.

3. Guidewires and catheters

Reasonable but approximated material properties were used to describe the behavior of the guidewires and catheters. Indeed, while it is essential to consider their deformability, the specific stiffness of these entities does not affect the simulation results. Thus, the geometries were defined based on the dimensions of the real parts, while the elastic modulus was established selecting average values coherent with the materials composition (Table 3B). More specifically, Nitinol for the guidewires, PTFE (Polytetrafluoroethylene) for the ID Branch delivery system, and a copolymer

composed of 50% of LDPE (Low-Density Polyethylene) and 50% of HDPE (High-Density Polyethylene) for the ID Cav delivery system.

TABLE 3B. Parameters used to define the guidewires and catheters behavior.

Material parameter	Units	Bench test	Guidewires	ID Cav catheters	ID Branch catheter
E	MPa	5	50000	675	450
v	-	0.45	0.3	0.45	0.45
Dext	mm	-	0.89	5.3	3.5
Dint	mm	-	-	4	3

Appendix C: Friction coefficients calibration

The friction coefficient defining the interaction of the stent with the crimping planes was calibrated based on the test on the ID Branch at 25°C. The value 0.05 allows obtaining a global mean difference with respect to the experimental data of 4.09%, exceeding the 10% only in the range 9.5-10mm. This deviation may be reduced by increasing the friction coefficient. Indeed, with 0.1, the differences within 9.5-10mm decrease below 10%, but they significantly increase in the range 0-9.5mm. Since the main interest is to predict the implanted configuration, which in general corresponds to an oversizing of 10-20% (diameter variation 1.4-2.8mm), the value 0.05 was selected.

Differences with respect to experimental results [%]				
Friction coefficient	0-9.5 mm Maximum	0-9.5 mm Mean	9.5-10 mm Maximum	9.5-10 mm Mean
0	11.75	5.21	20.64	18.18
0.05	7.47	3.38	14.71	13.15
0.1	11.89	6.40	9.33	5.70

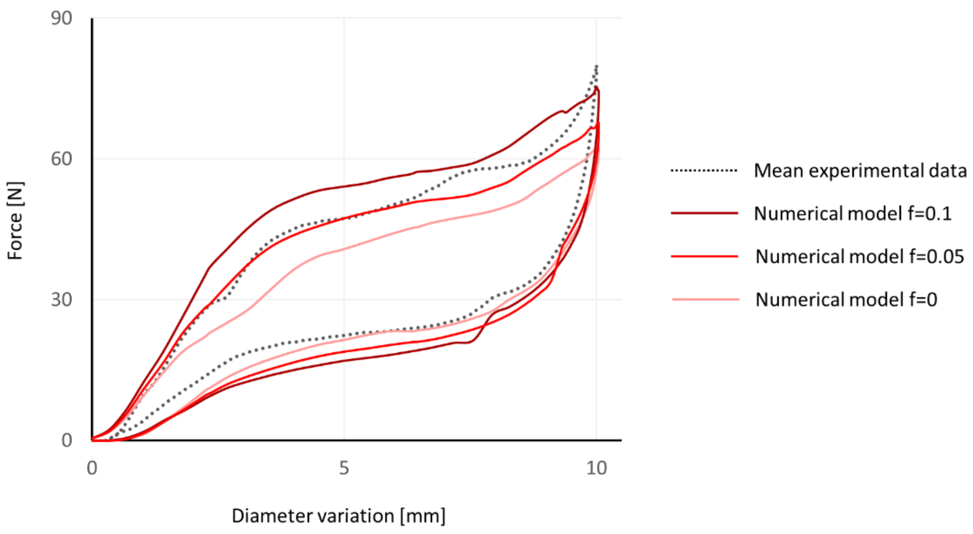

FIGURE 1C. Comparison among three friction coefficients (0, 0.05, 0.1) defining the interaction between the ID Branch and the crimping planes in terms of percentage difference with respect to the mean experimental data in the range 0-9.5mm and 9.5-10mm (top), and force-displacement curves superimposition (bottom).

The friction coefficient defining the interaction of the stent with the catheter was calibrated based on the release and recapture test of the ID Branch protrusion.

The value 0.03 was selected, given the accordance with the experimental data. Indeed, if the mean and maximum distance between the release and recapture curves is compared, the deviations with respect to the mean experimental data are 1.22% and 1.53%, respectively. These differences exceed 20% increasing or reducing the friction coefficient of 0.01.

Difference between the release and recapture curves				
Friction coefficient	Mean [N]	Error with respect to Experimental [%]	Maximum [N]	Error with respect to Experimental [%]
0.02	7.67	-30.08	12.34	-21.54
0.03	11.10	+1.22	15.48	-1.53
0.04	14.76	+34.62	19.69	+25.25
Experimental	10.96		15.72	

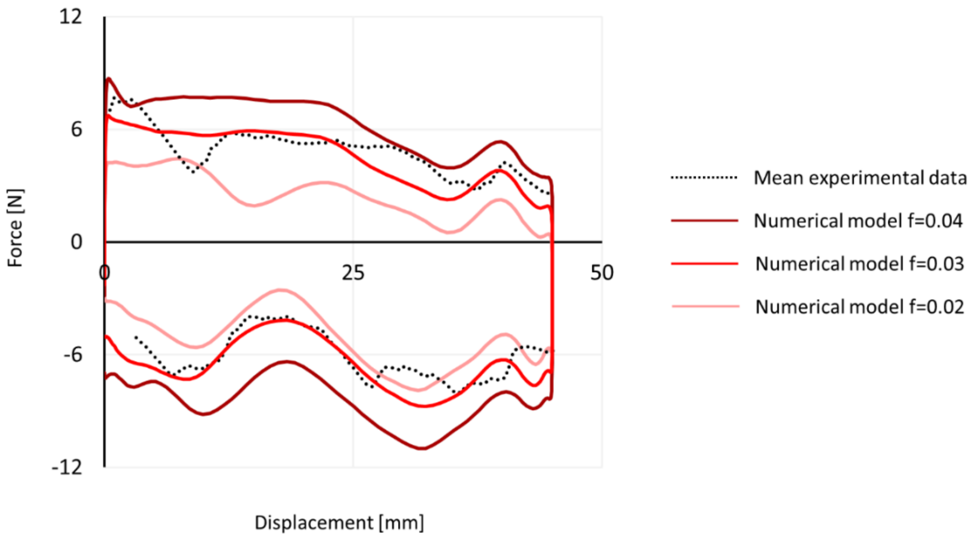

FIGURE 2C. Comparison among three friction coefficients (0.02, 0.03, 0.04) defining the interaction between the ID Branch and the catheter in terms of maximum and mean distance between the release and recapture curves (top), and force-displacement curves superimposition (bottom).

Appendix D: about experimental mean curves and Butterworth filter

In Figure 1D and 2D the force-displacement experimental curves for each specimen was reported with the related mean.

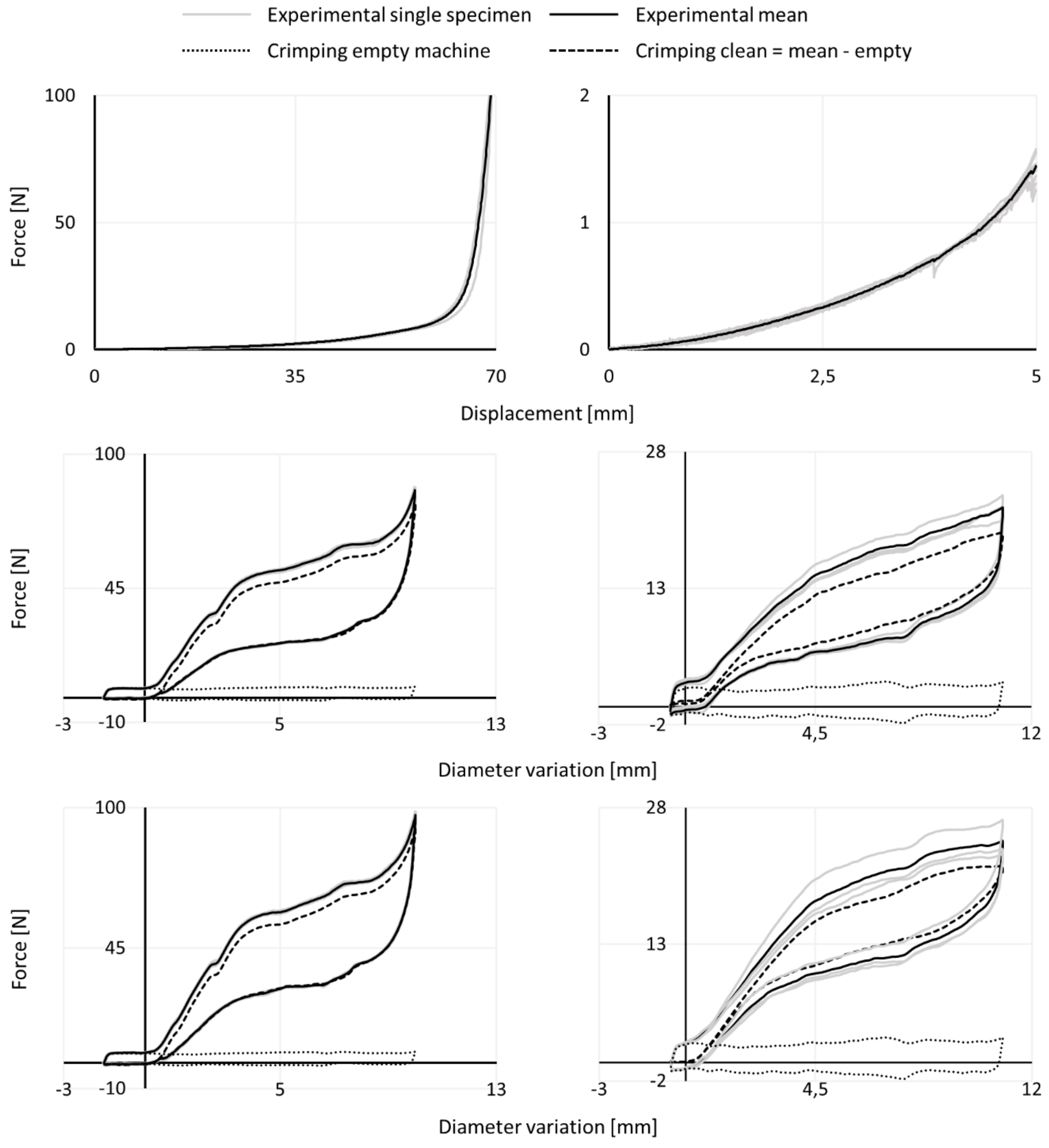

FIGURE 1D. Individual components behavior: force-displacement curves for each specimen and the related mean.

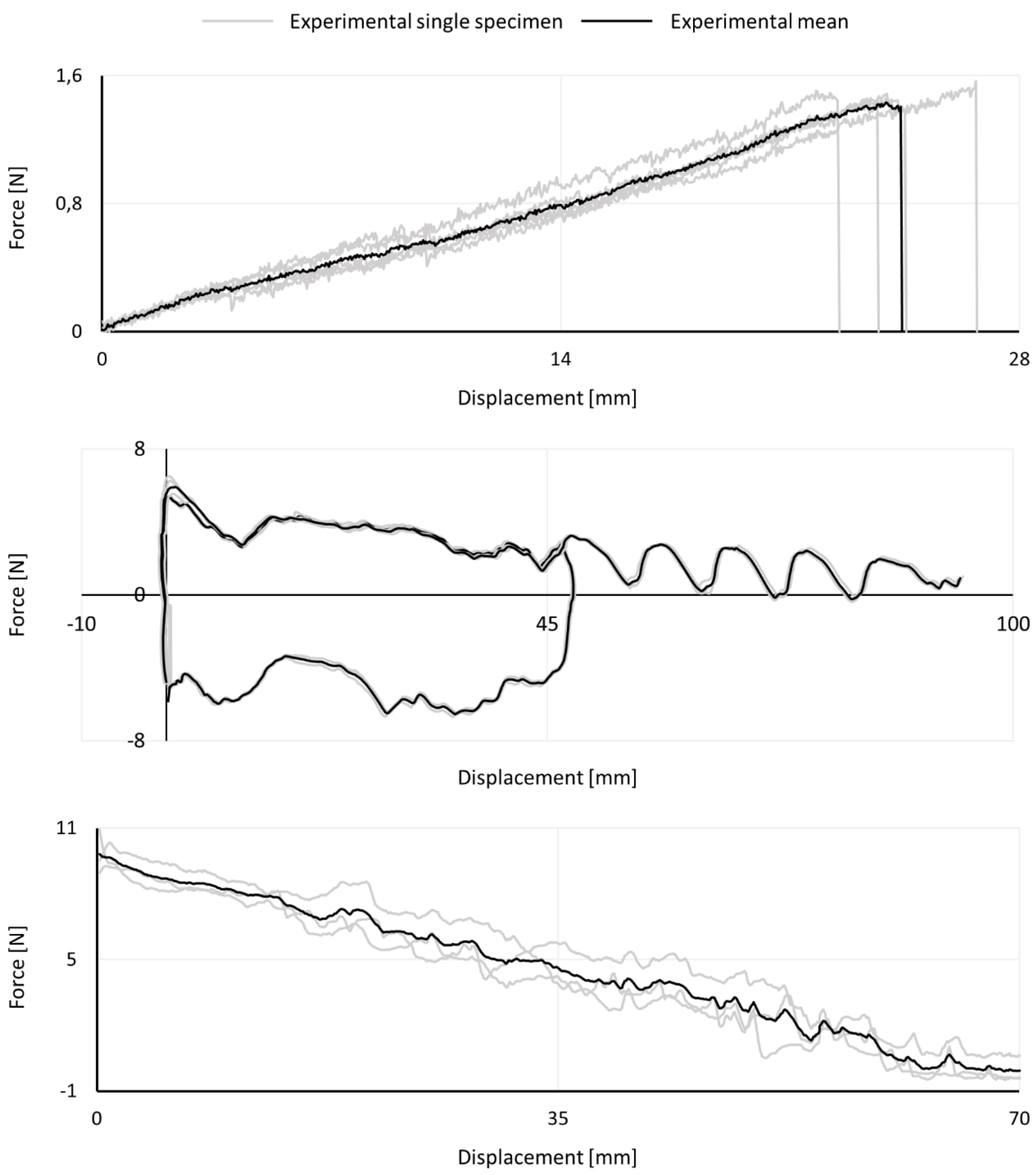

FIGURE 2D. Assembled system behavior and free-release tests: force-displacement curves for each specimen and the related mean.

In Figure 3D the original force-displacement numerical curves are compared with the filtered results for the free release tests. The maximum oscillations appear in correspondence of the ID Branch double twisted traits release (≈55.8 mm, ≈64.6 mm, ≈73.5 mm, ≈82.1 mm, ≈91.6 mm), probably due to approximation in the wires contact detection.

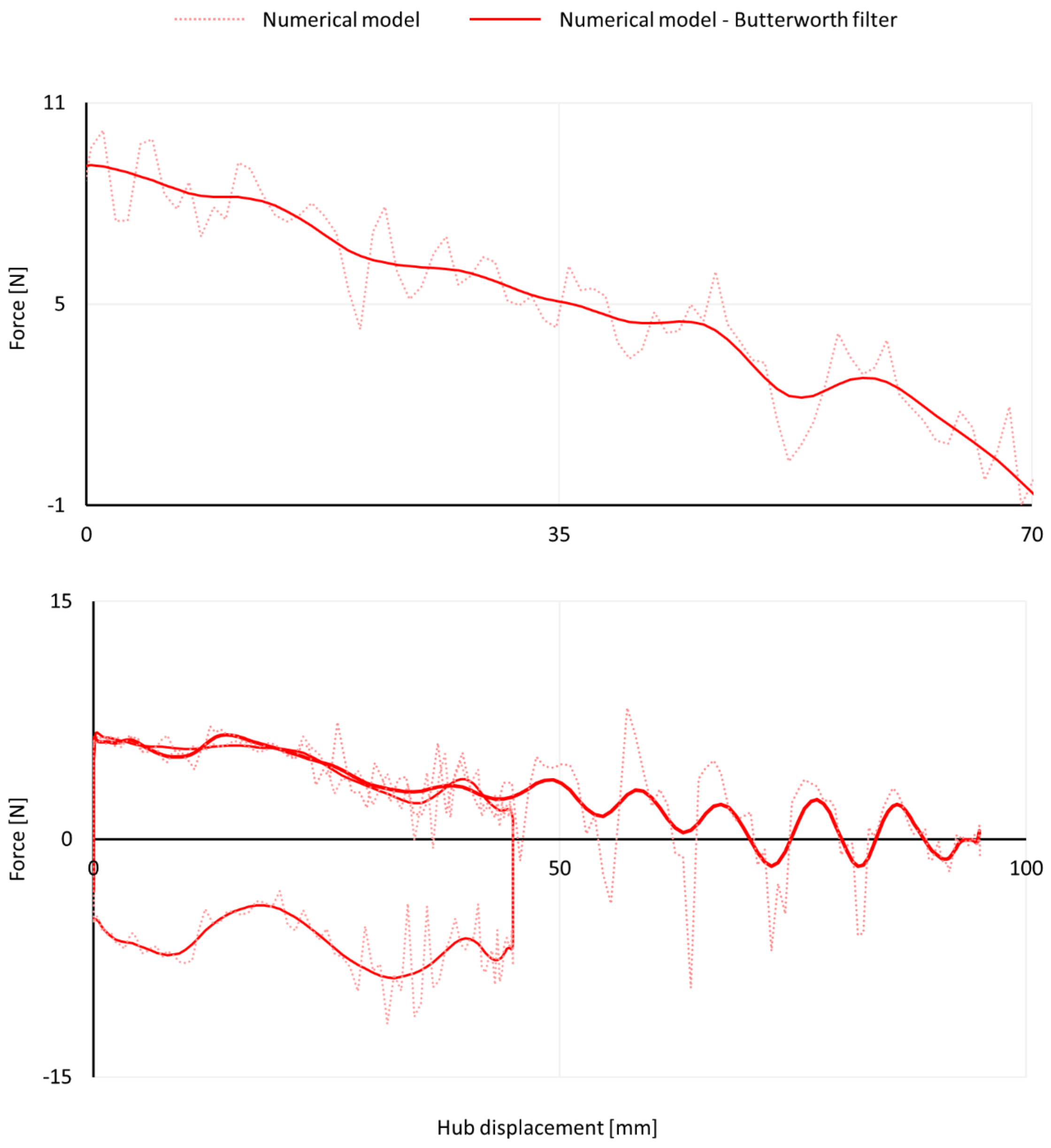

FIGURE 3D. Comparison between original and filtered force-displacement curves for the ID Cav (top) and ID Branch (bottom) free release tests.

REFERENCES

1. Allegretti, D., F. Berti, F. Migliavacca, G. Pennati, and L. Petrini. Fatigue Assessment of Nickel–Titanium Peripheral Stents: Comparison of Multi-Axial Fatigue Models. *Shape Mem. Superelasticity* 4:186–196, 2018.

2. Zaccaria, A., F. Danielli, E. Gasparotti, B. M. Fanni, S. Celi, G. Pennati, and L. Petrini. Left atrial appendage occlusion device: development and validation of a finite element model. *Med. Eng. Phys.* , 2020.doi:10.1016/j.medengphy.2020.05.019
